# Toward Understanding the Built-in Field in Perovskite Solar Cells through Layer-by-Layer Surface Photovoltage Measurements

**DOI:** 10.1021/acsami.4c14194

**Published:** 2025-02-05

**Authors:** Emilio Gutierrez-Partida, Marin Rusu, Fengshuo Zu, Meysam Raoufi, Jonas Diekmann, Nurlan Tokmoldin, Jonathan Warby, Dorothee Menzel, Felix Lang, Sahil Shah, Safa Shoaee, Lars Korte, Thomas Unold, Norbert Koch, Thomas Kirchartz, Dieter Neher, Martin Stolterfoht

**Affiliations:** † Institute of Physics and Astronomy, University of Potsdam, Karl-Liebknecht-Str. 24-25, D-14476 Potsdam-Golm, Germany; ‡ Department Structure and Dynamics of Energy Materials, Helmholtz-Zentrum-Berlin, Hahn-Meitner-Platz 1, D-14109 Berlin, Germany; § Humboldt-Universitat zu Berlin, Institut für Physik & IRIS Adlershof, Brook-Taylor Straße 6, D-12489 Berlin, Germany; ∥ Department Perovskite Tandem Solar Cells, Helmholtz-Zentrum-Berlin, Kekuléstr. 5, D-12489 Berlin, Germany; ⊥ IMD-3 Photovoltaics, Forschungszentrum Jülich GmbH, Wilhelm-Johnen-Straße, 52428 Jülich, Germany; # Faculty of Engineering and CENIDE, University of Duisburg-Essen, Carl-Benz-Str. 199, 47057 Duisburg, Germany; ∇ Electronic Engineering Department, The Chinese University of Hong Kong, Sha Tin N.T., Hong Kong SAR, China; $ Department Hybrid Material Systems, Helmholtz-Zentrum Berlin, Albert-Einstein-Straße 15, 12489 Berlin, Germany

**Keywords:** perovskite solar cells, built-in voltage (*V*_BI_), surface photovoltage (SPV), work function (WF) measurements, quasi-Fermi level splitting (QFLS).

## Abstract

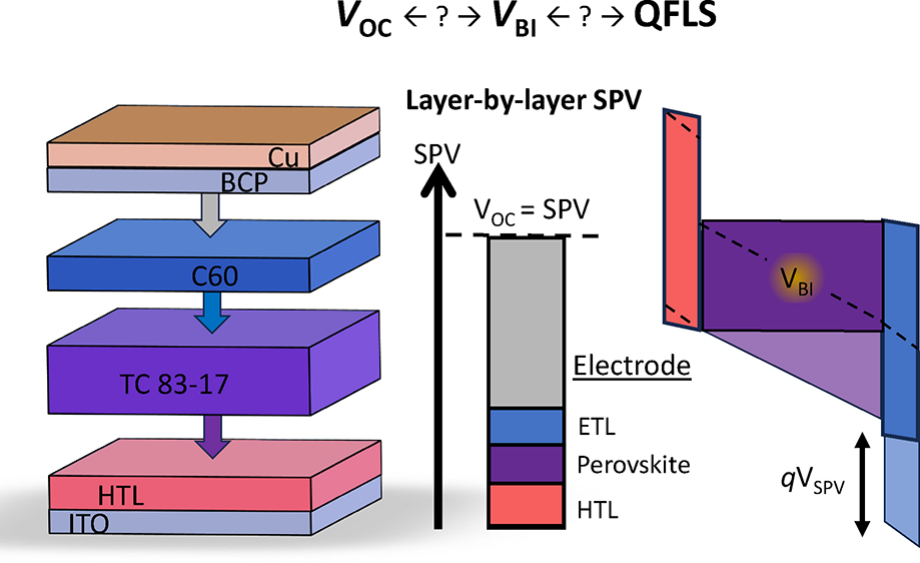

The built-in voltage (*V*_BI_) is a key parameter for solar cell operation, yet in perovskite solar cells the distribution, magnitude, and origin of the *V*_BI_ remains poorly understood. In this work, we systematically studied the *V*_BI_ in *pin*-type perovskite solar cells based on different hole transport layers (TLs). To this end, we determine the surface photovoltage (SPV) of partial and complete device stacks layer-by-layer by measuring the work function (WF) under dark and light (equivalent AM1.5G) conditions with Kelvin probe (KP) and photoemission spectroscopy (UPS) measurements in 3 different laboratories. We demonstrate that the SPV increases upon the addition of each additional layer until it equals the open-circuit voltage (*V*_OC_) of the full device. This suggests that both the electron and hole transport layer (HTL/ETL) enlarge the SPV, by improving the separation of photogenerated carriers. Yet, the contribution of both transport layers to the total SPV of the device is small (in the range of ≈100 to 200 meV) and the largest contribution to the SPV originates from the top metal electrode (≈500 meV). The results suggest that the *V*_BI_ of *pin*-type perovskite solar cells is largely a result of the work-function difference of the electrodes. With regard to films (or incomplete cell stacks), our simulations can reproduce the measured SPV, and measured quasi-Fermi level splitting (>*V*_OC_) in partial cell stacks without a significant internal field consistent with the experimental data. This work establishes layer-by-layer SPV measurements, which are easily accessible, as a key tool for understanding device performance and internal energetics, similar to layer-by-layer QFLS measurements.

## Introduction

Perovskite solar cells (PSCs) are attracting attention as a renewable energy source due to their low cost,^1^ high efficiency, and operational stability.^2−4^ Perovskite materials processed by low-cost solution-based methods have successfully achieved single junction solar cells with power conversion efficiencies (PCE) of >26% point conditions and 1 sun illumination and a range of standardized International Electrotechnical Commission (IEC) tests, making them an intriguing candidate for future PV technologies.^5^ In the past decade, various strategies have demonstrated their effectiveness in the process of device performance improvement, such as the optimization of device preparation methods, composition engineering of halide perovskites, and a wide range of interfacial modifications.

However, considering the past development of PSCs, gaining a comprehensive understanding of the built-in voltage (*V*_BI_) in complete PSCs has not been given sufficient attention despite its high importance for the open-circuit voltage (*V*_OC_), the fill factor (FF) and the complete device performance. The built-in voltage is an electrostatic potential difference between two contacts of the solar cell in the dark (i.e., the total voltage drop in the device across all layers) that for nearly every solar cell design makes a substantial contribution to the selectivity of charge carriers. Thus, the absence of a *V*_BI_ implies a lack of selectivity^6^ and typically produces losses related to the recombination of electrons at the hole contact (and vice versa) as well as barriers for the extraction of majority carriers that very often lead to S-shaped current–voltage curves.^7^ A high *V*_BI_ can also relax the requirements for high mobility in absorber^8,9^ or contact layers^10^ and can reduce recombination losses at short-circuit or the maximum power point due to inefficient charge extraction in solar cells with low conductivity layers.

**Figure 1:**
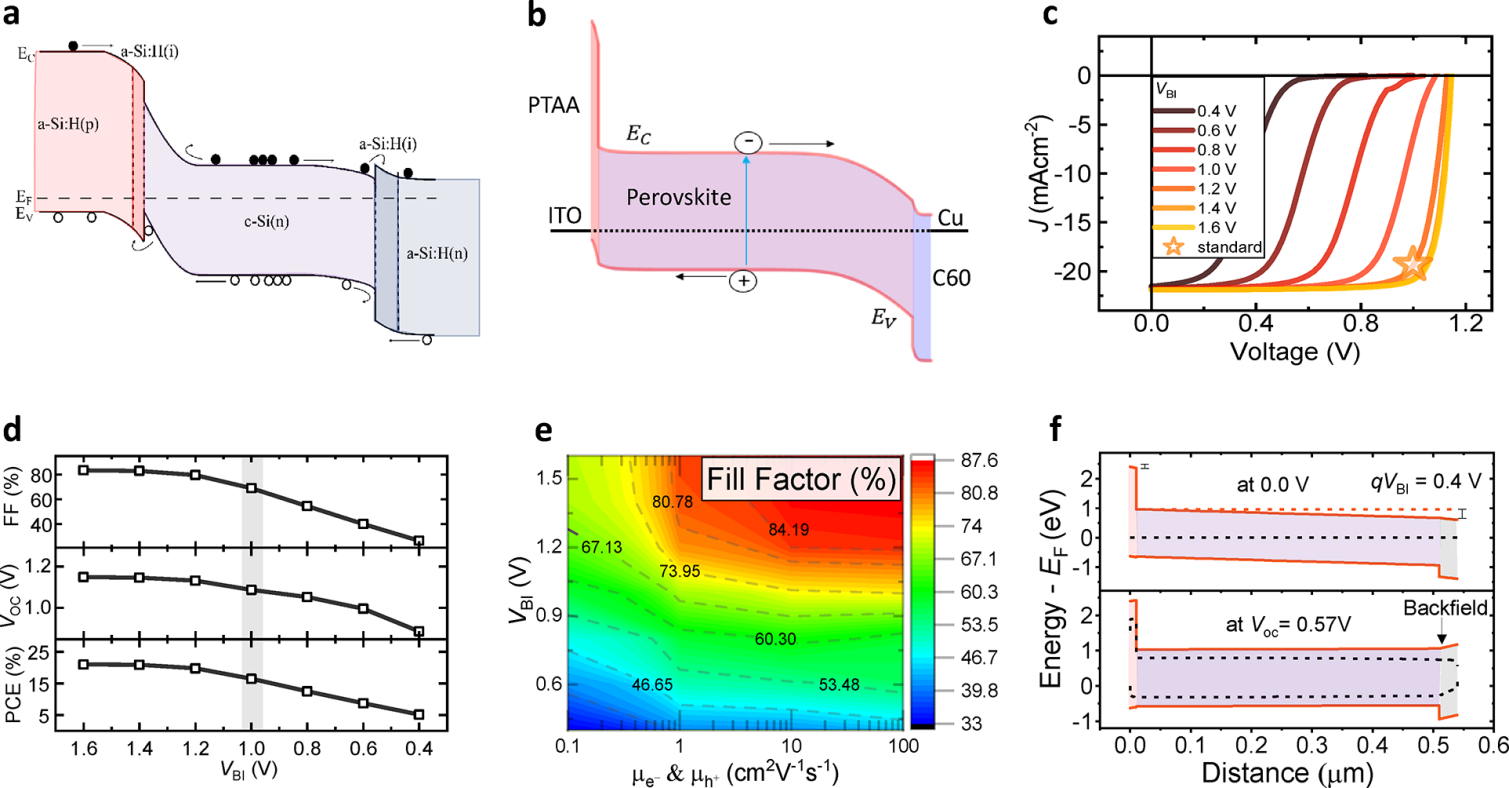
(a) Schematic energy band diagram of a heterojunction silicon solar cell. (b) Typical schematic band diagram of a generic perovskite solar cell. (c) *JV* characteristics and (d) illuminated *JV* parameters of a simulated *pin*-type perovskite solar cells demonstrating a relatively low fill factor (<70%) for cells with a *V*_BI_ lower than 1.2 V. (e) Heat maps of simulated power conversion efficiency (PCE) vs built-in voltage and electron and hole mobility in the perovskite layer (μ_e–_ and μ_h+_). In order to reproduce the experimental fill factor of ∼80%, a considerable *V*_BI_ of above 1.1 V is required regardless of the perovskite mobility because interface or bulk recombination deteriorates the FF in case of low driving voltages. (f) Simulated band diagrams showing the formation of an extraction barrier upon illumination for the device with a *V*_BI_ of only 0.4 V at its *V*_OC_ of only 0.57 V. Panel (a) adapted from ref (18). © The authors of ref (18) (CC-BY 4.0). Panel (b) adapted from ref (19). © The authors of ref (19) (CC-BY 4.0). Panel (c–e) adapted from ref (17). © The authors of ref (17) (CC-BY 4.0).

Generally, the band diagram provides information about the internal electric fields and thus about the transport and recombination processes at the interfaces and inside the layers. Understanding it could therefore help to achieve a good charge extraction efficiency and FF and a high *V*_OC_. In the case of silicon solar cells, as exemplified in Figure 1a, and thin film solar cells, the band diagram is generally well understood. Figure 1b exemplifies a typical band diagram for perovskite PV,^11^ proposed by different groups without conclusive results yet. In addition, there are only a few publications that aimed to determine the band diagrams of complete cells under short-circuit conditions.^11−13^ Reasons for the difficulties in obtaining an accurate band diagram also include the presence of mobile ions in perovskite semiconductors, which can redistribute and thereby change how the electrostatic potential changes between the anode to cathode. In addition, the combination of organic transport layers (TLs), metal oxides, and perovskite materials complicates measurements to assess the energy bands and the charge distribution.^14,15^ As such, it remains a major challenge to simulate experimentally measured band diagrams in perovskite solar cells while considering realistic interface and bulk recombination properties. Moreover, an analytical derivation of the band diagram based on a homogeneous field drop, the dielectric constants, and layer thicknesses is almost certainly prone to fail due to the presence of various interfacial phenomena, such as dipoles, the formation of junctions due to doped layers and mobile ions.^16^

With the help of a recently developed device model for PTAA/triple cation perovskite solar cells, we previously showed that a large *V*_BI_ is required in practical devices with nonzero bulk defects and recombination velocities (>1 cm/s) at the interfaces between the absorber and charge transport layers.^17^ The simulations displayed in Figure 1c–e show that a *V*_BI_ of at least 1 V needs to be present in order to simulate realistic *JV* curves, even for high mobilities in the perovskite. It is interesting to note that even in the absence of all nonradiative recombination in the bulk and at the interfaces (radiative recombination only), as well as mobilities of, e.g., 10 cm^2^/(Vs) in all layers (which is orders of magnitude larger than the mobilities in the organic transport layers),a significant *V*_BI_ is still required to reach high fill factors as shown in Figure S1. On another note, the presence of mobile ions in the bulk has opened up the debate on whether the bulk of the perovskite absorber can be field-free in a well-working device. It is well-known that mobile ion density in excess of the electrode charge density (*CV*_BI_, where *C* is the capacitance per surface area) will change the internal field distribution, although it is important to note that mobile ions do not cancel the given device *V*_BI_, they only redistribute it. In other words, while there might be field-free regions in the bulk of the absorber layer, the total voltage drop of all layers does not depend on the ion density. However, even if a field might not be required for efficient charge extraction from the perovskite, a *V*_BI_ across the device is still required to prevent the formation of a backfield when a voltage is generated, which would hinder the extraction of photogenerated charges. This is shown in Figure 1f. Lastly, we note that we investigated the intriguing question of whether a device with a larger mobile ion density could potentially sustain a lower device *V*_BI_ than we expected (i.e., less than 1 V) considering that the mobile ions could potentially screen the reverse field. However, the simulations shown in Figure S2 show that the presence of ions does not have a large beneficial effect at low *V*_BI_s even for near-ideal recombination properties. Although this requires further simulations, this further supports the assertion that efficient triple cation devices need to have a *V*_BI_ > 1 V. We note that the simulation parameters are shown in Table S1.

However, until today, it is unknown where the *V*_BI_ in PSCs originates from and how large it is. There are 3 main hypotheses. (1) The first possibility is that it could come from the perovskite itself, the formation of charged surface defects could create a space charge region at the surface of the perovskite.^20^ (2) The second possibility is that the *V*_BI_ originates from doped transport layers with different Fermi levels, i.e., (*E*_F,e,ETL_–*E*_F,h,HTL_)/*e*, where *E*_F,e,ETL_ and *E*_F,h,HTL_ are the majority Fermi level in the ETL and the HTL before contact formation, respectively. In fact, the use of doped TL to create a *V*_BI_ has the advantage that the whole voltage drops over the active layer. This suppresses interface recombination for given interfacial recombination velocities (*S*).^17^ (3) The third possibility is that the work function (WF) difference between the contacts is responsible for the device *V*_BI_ which is expected to be the case for OPV devices.^21^ A stronger field inside the perovskite can lead to faster extraction of charges and, therefore less recombination in the bulk and at the interfaces/contacts. However, until today and to our knowledge, there is no clear evidence that the effective work function of both contacts differs significantly from incomplete devices. It is important to mention that the work function of the buried contact might not be accessible after depositing a TL (e.g., HTL in *pin*-type cells). For example, materials such as Cu have shown a large work function shift when BCP was deposited on top (e.g., 0.6 eV shift in case of Cu).^22,23^ As such determining from which interface or layer the largest contribution of the *V*_B_ originates will provide us with a much clearer picture of the device physics of perovskite cells.

Another important aspect is that there is no generic and reliable method to determine the total *V*_BI_ in perovskite solar cells. For example, the Mott–Schottky analysis, which is often employed to determine the *V*_BI_, only provides a built-in voltage within the depletion approximation, which is typically not valid in the fairly intrinsic and thin perovskite solar cells.^14^ Instead features that can easily be mistaken for a built-in voltage are created by the capacitance response caused by injection of carriers from the contacts.^24^ Moreover, multiple device and material parameters strongly influence the capacitance and the *V*_BI_. Another tool that can help to understand the *V*_BI_ is surface photovoltage (SPV) measurements.^24^ The method determines the shift of the surface electrostatic potential between measurements taken in the dark and under illumination.^20,25−27^ Under illumination, photogenerated carriers are created due to band-to-band (or trap-to-band) transitions. These carriers will redistribute until previously existing gradients of the electrochemical potential between the front and the back of the sample have vanished. Therefore, the SPV is a common method to assess (approximate) the degree of band bending of semiconductors, including halide perovskites and it can be measured either with Ultraviolet Photoelectron Spectroscopy (UPS) or by Kelvin Probe (KP) as shown for example in refs (28,29). It is important to note that the term SPV does not necessarily imply that the photovoltage originates from the surface, but that it is measured at the surface. For PTAA/triple cation perovskite stacks, we note that large SPVs (close to 1 V) were observed with UPS measurements. This has been attributed to surface band bending due to Pb(0) states on the surface,^20^ although band bending at the buried interface can, in principle, also play a role.

In this work, we use layer-by-layer SPV measurements on films and cells to obtain a qualitative understanding of the contribution of each layer to the device *V*_BI_. To this end, we studied 5 different solar cells with different HTLs and different *V*_OC_s by systematically measuring KP in two different laboratories and UPS in a third lab on the same set of samples to reveal the contributions of the *V*_BI_ on each stack. We found that the SPV increases sequentially layer-by-layer. We also found that the *V*_BI_ is relatively small for partial stacks (<0.5 eV) consistent with previous works.^12,27^ Nevertheless, a large QFLS is present in the films as measured from photoluminescence (PL). However, translating this QFLS into the open-circuit voltage of a device can only be achieved in the case of a relatively large *V*_BI_ (>1 V) in our device. Using drift-diffusion simulations, we are able to reproduce the measured SPV and QFLS values layer-by-layer. This not only corroborates our results but highlights new possibilities to improve the accuracy of device models of perovskite solar cells in the future. Our measurements reveal that the required *V*_BI_ in efficient *pin*-type solar cells originates to a large degree from electrodes with different work functions.

## Experimental Section, Methods and Materials

### Device Fabrication

Prepatterned 2.5 × 2.5 cm^2^ 15 Ω/sq. ITO (Automatic Research, Germany), glass or fused silica substrates were cleaned with acetone, 3% Hellmanex solution, DI-water and *iso*-propanol, by sonication for 10 min in each solution. Afterward a microwave plasma treatment (4 min, 200 W) was performed for the NoHTL, PEDOT:PSS, P3HT, PolyTPD and PTAA devices. The samples were transferred to an N_2_-filled glovebox (except PEDOT:PSS which was spin-coated in air. For the SAM device, the same ITO coated samples were used but instead of the microwave plasma treatment, the substrates were treated with ultraviolet ozone (UVOzone) for 30 min. The samples were then transferred to a nitrogen-filled glovebox and SAM-2PACz purchased from TCI (1 mmol mL^–1^ in ethanol) was spin-coated on the ITO substrates at 3000 rpm for 30 s, followed by annealing at 100 °C for 10 min.

#### Bottom Selective Contacts

(HTLs): PEDOT:PSS (Heraeus Celivious 4083) was spin-coated at 4000 r.p.m for 40s (acceleration 2000 r.p.m/s) and subsequently annealed at 150 °C for 15 min; P3HT (Sigma-Aldrich, *M*_n_ ∼ 27,000) was spin-coated from a 2 mg/mL DCB solution at 3000 r.p.m for 30s (acceleration 3000 r.p.m/s) and subsequently annealed 100 °C for 10 min. P3HT films were also oxygen plasma treated for 5 s to ensure sufficient wetting of the perovskite as discussed in a previous work.^30^ PolyTPD (Ossila) was spin-coated from a 1 mg/mL Toluene solution at 6000 r.p.m for 30 s (acceleration 2000 r.p.m/s) and subsequently annealed 100 °C for 10 min. PTAA (Sigma-Aldrich) was spin-coated from a 2 mg/mL Toluene solution at 6000 r.p.m for 30 s (acceleration 2000 r.p.m/s) and subsequently annealed 100 °C for 10 min. For PTAA coated samples, a 60 μL solution of PFN-P2 (0.5 mg/mL in methanol) was added onto the spinning substrate at 5000 rpm for 30 s resulting in a film with a thickness below the detection limit of our AFM (<5 nm). SAM-2PACz (TCI) was spin-coated from a 1.0 mg/mL Ethanol solution at 3000 r.p.m for 30 s (acceleration 3000 r.p.m/s) and subsequently annealed 100 °C for 10 min.

#### Perovskite Layer

The triple cation perovskite solution was prepared by mixing two 1.3 M FAPbI_3_ and MAPbBr_3_ perovskite solutions in DMF:DMSO (4:1) in a ratio of 83:17 which we call “MAFA” solution. The 1.3 M FAPbI_3_ solution was thereby prepared by dissolving FAI (722 mg) and PbI_2_ (2130 mg) in 2.8 mL DMF and 0.7 mL DMSO (note there is a 10% excess of PbI_2_). The 1.3 M MAPbBr_3_ solution was made by dissolving MABr (470 mg) and PbBr_2_ (1696 mg) in 2.8 mL DMF and 0.7 mL DMSO (note there is a 10% excess of PbBr_2_). Lastly, 40 μL of a 1.2 M CsI solution in DMSO (389 mg CsI in 1 mL DMSO) was mixed with 960 μL of the MAFA solution resulting in a final perovskite stoichiometry of (CsPbI_3_)_0.05_[(FAPbI_3_)_0.83_(MAPbBr_3_)_0.17_]_0.95_ in solution. The perovskite film was deposited by spin-coating at 4000 r.p.m (acceleration 1300 rpm/s) for 40 s; 13 Seconds after the start of the spinning process, the spinning substrate was washed with 300 μL EA for approximately 1 s (the antisolvent was placed in the center of the film). The perovskite film was then annealed at 100 °C for 1 h on a preheated hot plate.

#### Top Selective Contacts

(ETLs): After annealing, the samples were transferred to an evaporation chamber where fullerene-C60 (Sigma-Aldrich, 30 nm) and 2,9-Dimethyl-4,7-diphenyl-1,10-phenanthroline BCP (Sigma-Aldrich, 8 nm) were deposited under vacuum (*p* = 10^–7^ mbar).

#### Metal Contacts

Similarly to the ETL, 100 nm copper (Sigma-Aldrich) at 0.6 Å/s were deposited under vacuum (*p* = 10^–7^ mbar). The overlap of the copper and the ITO electrodes defined the active area of the pixel (6 mm^2^).

### Current Density–Voltage Characteristics

*JV*-curves were obtained in a 2-wire source-sense configuration with a Keithley 2400. An Oriel class AAA xenon lamp-based sun simulator was used for illumination providing approximately 100 mW cm^–2^ of AM1.5G irradiation and the intensity was monitored simultaneously with a Si photodiode. The exact illumination intensity was used for efficiency calculations, and the simulator was calibrated with a KG3 filtered silicon solar cell (certified by Fraunhofer ISE). The device area of 6 mm^2^ was masked with a 4.32 mm^2^ mask and the obtained short-circuit current densities (*J*_SC_) were checked by integrating the product of the External Quantum Efficiency and the solar spectrum which matches the obtained *J*_SC_ within less than 5%. The temperature of the cell was fixed to 25 °C and a voltage ramp (scan rate) of 67 mV/s was used. *JV*-curves at different scan speeds and stabilization times are subject of this work. For the 83–17 reference cell, a spectral mismatch calculation was performed based on the spectral irradiance of the solar simulator, the EQE of the reference silicon solar cell and 3 typical EQEs of our cells. This resulted in 3 mismatch factors of *M* = 0.9949, 0.9996, and 0.9976. Given the very small deviation from unity the measured *J*_SC_ was not corrected by the factor 1/*M*.

### Numerical Drift-Diffusion Simulations

The simulations were performed using SCAPS which numerically solves a system of three coupled equations, namely the Poisson equation, the continuity equation and the drift-diffusion equation.^17^

### Absolute Photoluminescence Measurements

Excitation for the PL measurements was performed with a 520 nm CW laser (Insaneware) through an optical fiber into an integrating sphere. The intensity of the laser was adjusted to a 1 sun equivalent intensity by illuminating a 1 cm^2^ -size perovskite solar cell under short-circuit and matching the current density to the *J*_SC_ under the sun simulator for the 83–17 triple cation device (22.0 mA/cm^2^ at 100 mW cm^–2^, or 1.375 × 10^21^ photons m^–2^ s^–1^). A second optical fiber was used from the output of the integrating sphere to an Andor SR393iB spectrometer equipped with a silicon CCD camera (DU420A-BR-DD, iDus). The system was calibrated by using a calibrated halogen lamp with specified spectral irradiance, which was shone into to integrating sphere. A spectral correction factor was established to match the spectral output of the detector to the calibrated spectral irradiance of the lamp. The spectral photon density was obtained from the corrected detector signal (spectral irradiance) by division through the photon energy (*hf*), and the photon numbers of the excitation and emission obtained from numerical integration using Matlab. In a last step, three fluorescent test samples with high specified PLQY (∼70%) supplied from Hamamatsu Photonics where measured where the specified value could be accurately reproduced within a small relative error of less than 5%.

#### Photoemission Spectroscopy

Ultraviolet photoelectron spectroscopy measurements were performed at a SPECS ultrahigh vacuum system (base pressure of 5 × 10^–10^ mBar) using a monochromatized helium discharge lamp (21.22 eV). The sample work function is directly determined from the secondary electron cutoff (SECO) spectra, which were recorded at a bias of −10 V on the samples to overcome the work function of the analyzer. The photoemission system was calibrated by setting the Fermi edge of an Au sample at 0 eV binding energy. All spectra were recorded at room temperature and normal emission. Sample illumination during SECO measurements was conducted using a white halogen lamp (Solux MR16 4700 K, daylight rendering, the emission spectrum can be obtained at www.solux.net/cgi-bin/tlistore/infopages/4700k.html#TechnicalSpecifications) at a controlled intensity of ca. 100 mW/cm^2^.

#### Kelvin Probe Measurements

WFs were measured in a N_2_-filled glovebox at room temperature with a KP Technology SKP5050 KP. Note that the KP setup was housed in a metal box, which reduced the electromagnetic noise but also protected the setup and the sample from light. The setup used at HZB had a minor difference: instead of being inside a nitrogen-filled glovebox, the measurement was conducted without air exposure of samples in a smaller (Faraday) box filled with nitrogen.

Kelvin probe (KP) and photoelectron yield spectroscopy (PYS) were combined to measure the work function (Φ) of the prepared thin films, respectively, by employing a KP Technology SKP5050-APS02 setup under N_2_ ambient. The KP system uses a 2.0 mm diameter tip with a gold alloy coating which is calibrated on a freshly cleaved, highly ordered pyrolytic graphite (HOPG), for which we assumed a WF of 4.6 eV. The KP is placed in a Faraday cage which screens the external electrical fields.

The work function was determined by measuring the contact potential difference (CPD) between the Kelvin probe (reference electrode) with a known work function, Φ_tip_, and the investigated thin film. The CPD is measured with a resolution of 3 mV. Since Φ_tip_ is known from reference measurements on an Au thin film, the work function of the sample, Φ_sample_, is calculated as^28^
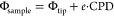
1where *e* is the elementary charge. The measurements of the surface photovoltage (SPV) defined as SPV = CPD_light_–CPD_dark_ were performed using a white light source with a spectrum of the blackbody at 5700 K and calibrated for the light power density of ∼100 mW/cm^2^. Further details on the KP, as well as the applied methodology of evaluating the experimental data can be found elsewhere.^28^

## Results

**Figure 2:**
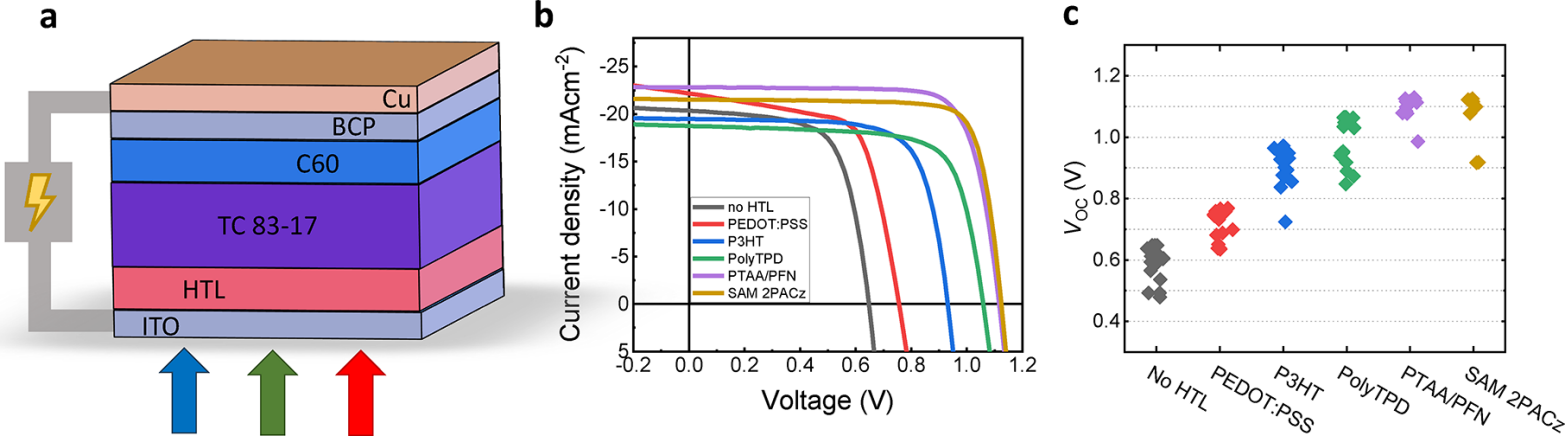
(a) Schematic of the standard *pin* perovskite solar cell used for all the stacks. (b) Representative *JV* curves of devices for different HTLs. (c) Trend in open-circuit voltage for solar cells with different hole transport layers.

In order to study the change in the *V*_BI_ due to the different stack layers and the effect of different HTLs, we have prepared devices with different HTLs in a *pin*-architecture shown in Figure 2a. The HTLs include indium tin oxide (ITO) in direct contact with the perovskite, which we referred to as “no HTL”, poly(3,4-ethylenedioxythiophene) polystyrenesulfonate (PEODT:PSS) referred to as “PEDOT”, Poly(3-hexylthiophen-2,5-diyl) referred to as “P3HT”, poly(N,*N*′-bis-4-butylphenyl-N,*N*′-bisphenyl)benzidine labeled as “PolyTPD”, poly[bis(4-phenyl)(2,4,6-trimethylphenyl)amine labeled as “PTAA” and finally 2-(9*H*-Carbazol-9-yl)ethyl]phosphonic acid labeled as “2PACz”. As absorber material, we used an “83:17 triple cation” (Cs_5_[FA_83_MA_17_]_95_)Pb[I_83_Br_17_]_3_) perovskite while the electron transport layer (ETL) was C_60_ and BCP. The electrodes were made of indium tin oxide (ITO) and copper. The implementation of these different HTLs leads to largely different *V*_OC_ values, potentially allowing us to investigate the impact of the SPV and *V*_BI_ on the *V*_OC_ and the QFLS of these devices.

The current–voltage characteristics of representative devices is shown in reverse scan direction measured with a scan speed of 67 mV/s in Figure 2b,c. We obtained an increase in the *V*_OC_ starting from 0.6 V for the “no HTL” sample until approximately 1.13 V for devices with PTAA and 2PACz. The *JV* curves show the measured performance for the different HTLs. The other photovoltaic performance metrics are shown in Figure S3. We note that we observed a low shunt resistance for devices with PEDOT:PSS as HTL (∼500 Ω cm^–2^), however, the simulations show that this has only a relatively small impact on the overall performance.

**Figure 3:**
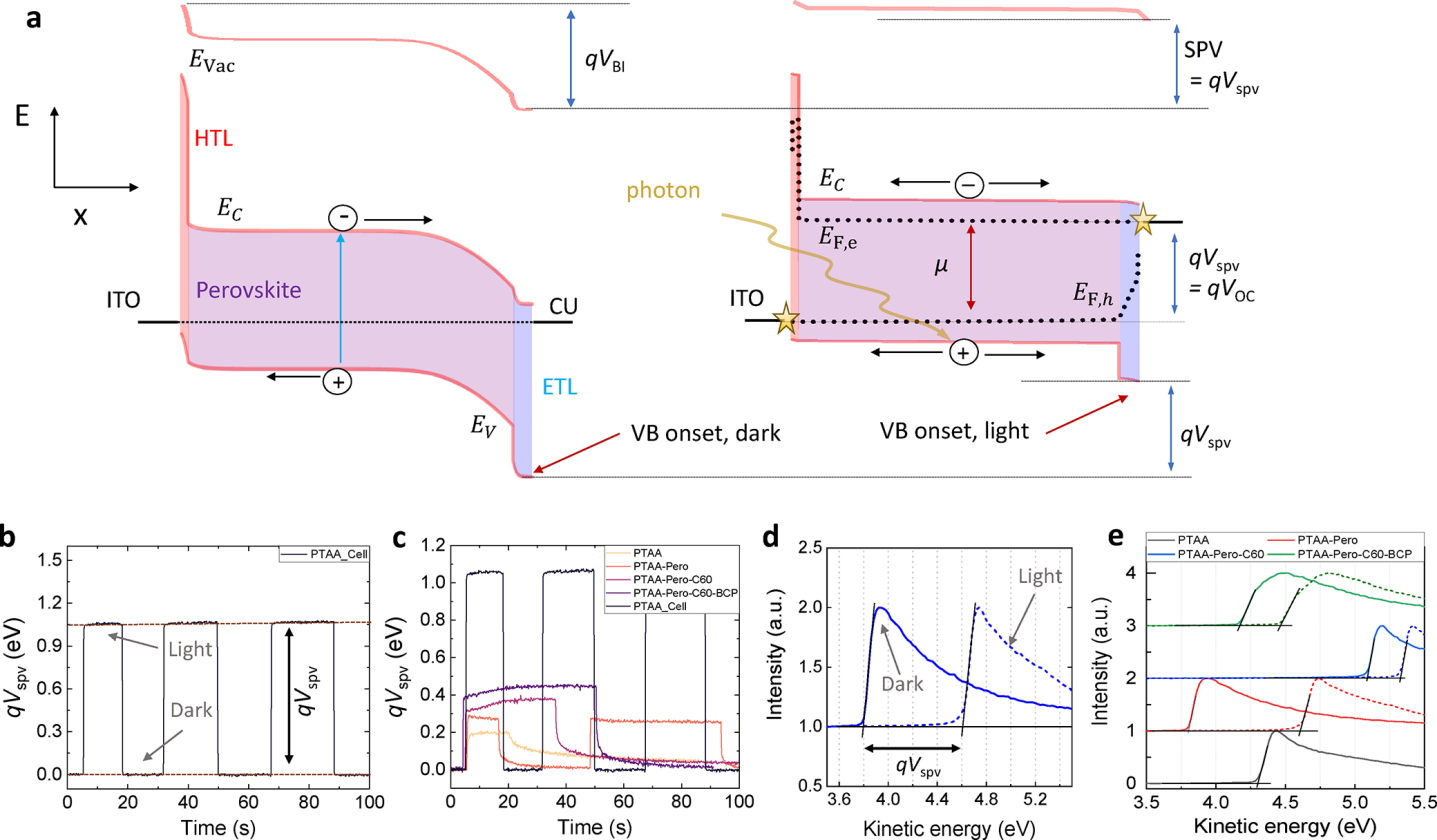
(a) Energy band diagrams schematic before and after illumination showing the surface photovoltage (SPV) effect of perovskite cells (valence band maximum *E*_V_ and conduction band minimum *E*_C_ and vacuum level *E*_Vac_) in the dark (left) and open-circuit (right). The HTL is depicted in red, the perovskite layer in purple, and the ETL in blue. The *V*_BI_ is defined as the total voltage drop across all layers of the device in the dark equilibrium. In the simulations and in the experiments (KP, UPS) all energy levels are referenced to the Fermi level of ITO. In this simulation, the internal voltage (*V*_BI_) is nearly compensated by photogenerated charges, and a QFLS is created. The hole QFL might be aligned with the ITO Fermi level in case of well-aligned energy levels. The generated SPV can be measured from the difference in the work function (*E*_Vac_ level with respect to the Fermi level) in the light and in the dark. Note that the position of the *E*_V_ shifts accordingly (as confirmed with UPS). The graph also shows that the *V*_OC_ equals the SPV by definition which is further equal to the QFL difference between the electron QFL in the ETL and the hole QFL in the HTL. Thus, the SPV is a measure of the voltage produced in the cell or partial cell stacks. (b) Exemplified transient KP measurement to illustrate how the SPV is quantified from the dark and light work function values. (c) Comparison of SPVs measured with KP for different stacks. (d) UPS spectra for half stacks under dark and light conditions illustrating the work function shift due to the SPV. (e) SPV obtained from UPS measurements of the work function for different stacks. Panels b, c, d and e measured on the “PTAA” device.

We now turn our attention to the identification of the origin of the *V*_BI_ via layer-by-layer SPV measurement on all devices using Kelvin probe and UPS measurements in 3 laboratories (KP at University of Potsdam (UP), KP at the Helmholtz-Zerntrum Berlin (HZB) and UPS at the Humboldt University Berlin (HU)). To this end, each lab was provided with pieces of the same sample (1 cm^2^) which was broken along precut lines on the substrate after spin coating of the corresponding layer. In the following, the results from UP are discussed while the results from the other laboratories are shown and compared in the Supporting Information. We will also discuss the lab-to-lab differences along with the discussion of the results. Figure 3a first demonstrates a schematic of the process happening in the device that causes the SPV effect (all details in the caption). Experimentally, the SPV with KP is measured by illuminating the film/device with a white LED (without UV light) with a 1 sun equivalent light intensity (where the generated current matches the *J*_SC_ under simulated AM1.5G). Corresponding traces of “no HTL” devices are shown Figure 3b,c for different layer stacks. Figure 3d shows the measured work function in the dark and in the light from UPS measurements for different stack layers for the “PTAA” device.

**Figure 4:**
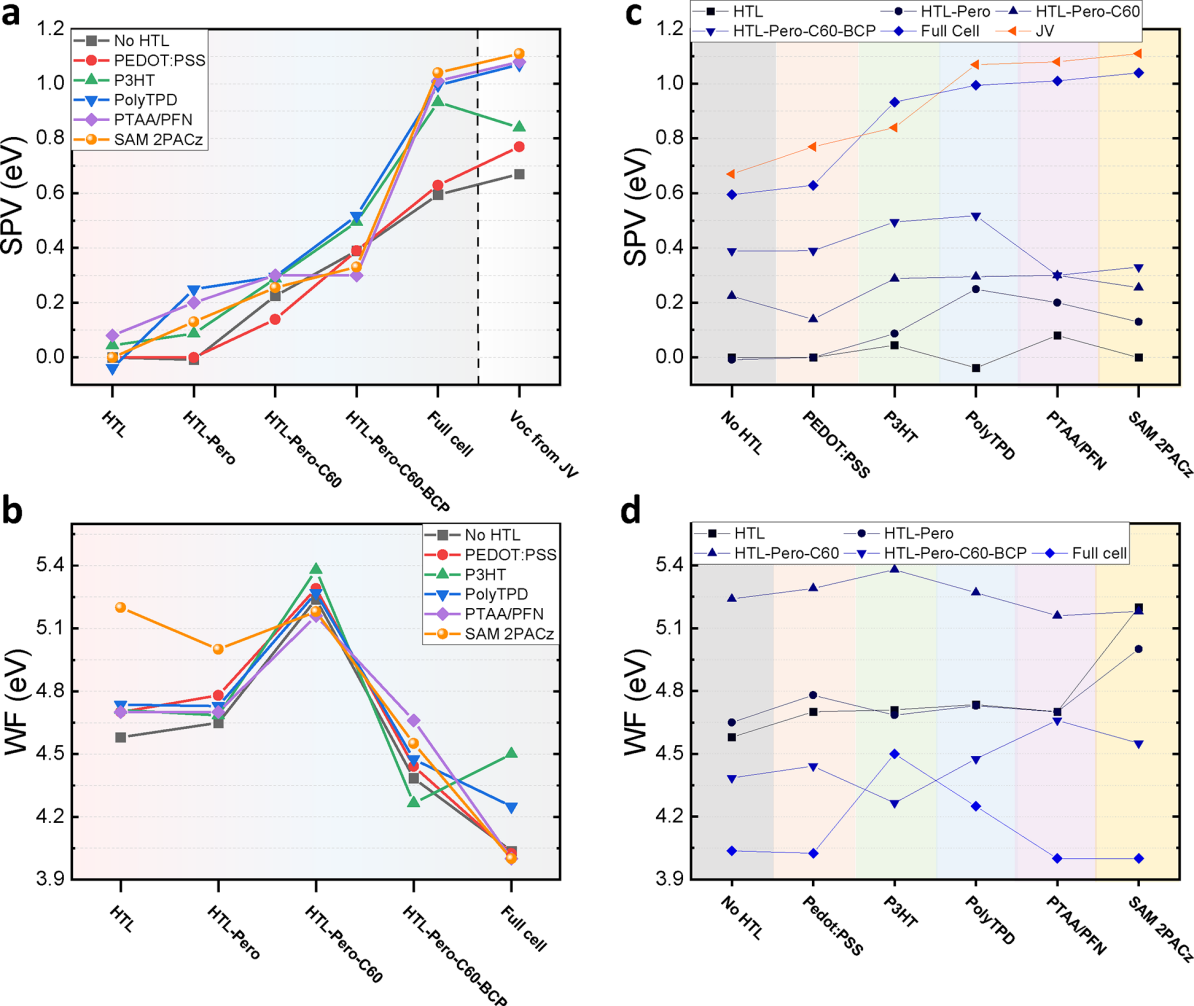
(a, c) Layer-by-layer surface photovoltage (SPV) and (b, d) work function values for each device in dark conditions. Panel (a) shows that the addition of each layer increases the SPV until the *V*_OC_ is reached for the complete stack which is also plotted for comparison. The work function follows a similar trend for all HTLs and the difference between the HTL work function and the Cu electrode is significant in all cases although it falls short of the expected built-in voltage (*V*_BI_) as discussed in Figure 1, with exception for the SAM device where a WF difference of 1.2 eV is seen.

The SPV results obtained from Kelvin probe measurements at UP on each stack layer are shown in Figure 4a,c, while the values are specified in Table 1 (while Figures S4–S7 and Tables S2 and S3 show the results from all laboratories). First, we found that overall each additional layer adds to the total SPV although the exact contribution depends on the TL. Starting from the ITO/HTL stacks, no SPV is observed as expected with the exception of PTAA (possibly due to a small junction between ITO and PTAA). We note that a TL with a large work function would in principal cause a larger SPV than a TL with a lower WF that is in middle of the perovskite bandgap as schematically illustrated in Figure S8.^31^ Interestingly, given that the SPV of the ITO/2PACz/perovskite stack is also very small despite the high *V*_OC_ of this device, we conclude that there is no clear correlation between the SPV and the optoelectronic quality of the HTL and the device *V*_OC_ in our devices. There’s ongoing discussion in the scientific community regarding the importance of the SPV of individual layers for the overall SPV of a device. For example ref (32) demonstrates a correlation between the SPV measured on individual layers and the overall device *V*_OC_, suggesting that optimizing individual layer SPV can be a strategy for enhancing overall device performance. Also ref (33) utilizes SPV measurements to investigate the impact of different contact materials on the performance of inverted perovskite solar cells, highlighting the value of SPV as a diagnostic tool for material and interface optimization. Going forward, we find that the addition of the ETL increases the SPV for all studied devices, either due to the increased selective separation of electrons (and holes) to the TLs or due to the formation of a band bending at the perovskite/C_60_ interface (potentially increasing the *V*_BI_), which is discussed in detail below. Also, the addition of BCP increases the SPV in all cases. This coincides with the drop of the WF of the BCP-coated C_60_, thus BCP likely enhances charge extraction even without the Cu layer. Interestingly, however, even after the addition of BCP deposition, the SPV remains relatively low in all cases, i.e., significantly below the *V*_OC_ of the devices. This indicates that there is actually no large *V*_BI_ present in the partial cell stacks as discussed below.

**Table 1:** SPV and *V*_OC_ Results Compared for Different HTLs and Stacks^a^

		No HTL	PEDOT:PSS	P3HT	PolyTPD	PTAA/PFN	2PACz
SPV [V]	HTL	0	0	0.04	–0.04	0.08	0
	HTL-Pero	–0.01	0	0.09	0.25	0.20	0.13
	HTL-Pero-C60	0.22	0.14	0.29	0.29	0.30	0.26
	HTL-Pero-C60-BCP	0.39	0.39	0.49	0.52	0.31	0.33
	complete device	0.59	0.63	0.93	0.99	1.01	1.04
*V*_OC_ [V]	*JV*	0.67	0.77	0.84	1.07	1.08	1.11

a Values for complete device SPV, *JV* and CV similar for each HTL (±50 mV) proving the accuracy of the measurements.

Finally, addition of the Cu top electrode significantly increases the SPV in all cases, even by ≥500 meV in the best-performing cells (PTAA, 2PACz, Figure S9). In addition, the SPV of the full cell agrees with the device *V*_OC_ reasonably well as expected, which confirms the validity of the measurements. The results demonstrate that the largest contribution to the *V*_BI_ comes from the completion of the device with the top/back electrode, indicating that the *V*_BI_ is mainly a result of (effectively) work function mismatched electrodes. We note the added SPV upon the Cu addition is smaller in the case of less efficient cells (“NoHTL”, and “PEDOT:PSS”) because the *V*_BI_ in those cells is likely reduced as a result of the HOMO_HTL_-VBM_pero_ offset. To highlight this effect, i.e., that an energy level misalignment would predominately affect the device *V*_OC_, rather than the SPV of the partial cell stacks, we have simulated the SPV layer-by-layer for the PEDOT:PSS sample as shown in Figure S10 (this will be discussed further below in more detail). The work function of each layer for all studied cells is further plotted in Figure 4b,d. The relatively low Cu work function of about 4 eV in the case of a 7 nm-thin Cu film on top of the ETL layer is interesting considering that the work function of bare Cu without other layers is around 4.7 eV depending on the crystal orientation (similar to the work function of ITO).^34−36^ We attribute this effect to Cu diffusion into the BCP layer which leads to a metal–organic complex with a much lower work function than bare copper.^22,23^ To further investigate this hypothesis, we compared a PTAA-based device w/and w/o a BCP layer and measured the Cu work function and we found that it is indeed lower w/the BCP layer underneath compared to the cell w/o the BCP layer (Figure S11a). Moreover, the FF of the cell w/o BCP is significantly lower compared to the cell with BCP (Figure S11b) which is consistent with the lower *V*_BI_ according to simulations (Figure S11c). However, it should be noted that the simulations could not exactly reproduce the device performance without BCP, meaning there is likely another factor that affects the performance when BPC is left out, which affects particularly the *J*_SC_. Nevertheless, this further indicates the importance of the combination of the BCP layer with the Cu film for lowering the cathode work function to increase the device *V*_BI_.

Figure 4b,d also reveals that the work function as measured on the top surface of each layer does not follow the trend of the CB and VB. In particular, no monotonic “downwards” trend from the left (*p*-side) to the right (*n*-side) is observed as shown in Figure 3a. We note that the increasing WF with C_60_ thickness is likely due to the high electron affinity of the fullerene and thus the WF increases until a full monolayer is reached above several nm of C_60_ (Figure S12).^37^ It is also important to note that the high WF of C_60_ is not detrimental to the device’s performance because it is the alignment of the highest occupied and lowest unoccupied energy level that is important for efficient transport of charges. Moreover, it is the difference between the (effective) work functions of the contacts that is essential for the *V*_BI_. Figure 4b,d shows that in case of the 2PACz device, a large difference between the ITO/SAM and Cu of nearly 1 V is observed which is consistent with the lower bound of the expected *V*_BI_ (Figure 3). However, for all other HTLs, the work function difference between ITO/HTL and Cu is observed to be lower (∼0.6 V) than the expected *V*_BI_ which requires further investigation.

With regard to the results obtained by the other laboratories, as shown in Figures S4–S7, Kelvin probe results between both laboratories matched qualitatively well. However, UPS shows a significantly higher SPV in the case of ITO/PolyTPD (∼0.6 V) or PTAA/perovskite (∼0.8 V) stacks, which decreases after the addition of the ETL (∼0.25 V). Although the reason is not clear this might be related to the specific measurement conditions during the UPS measurement, which might affect the results obtained on PTAA/triple cation samples. Moreover, we note that complete devices could not be assessed with UPS as the devices degraded during the measurement or during the postage of the samples.

In the following, we aim to reach a quantitative description of the SPV results for the reference PTAA/triple cation device using numerical simulations. We further take the measured QFLS for each stack layer into account. To this end, we performed drift-diffusion device simulations using again the software SCAPS, which was developed at the University of Gent,^38^ as well as SETFOS from FluXim for simulations with ions.^39^ To simulate the partial cell stacks, we assume that a relatively small field (e.g., 0.2 eV) is present in the partial cell stacks (because the *V*_BI_ is mainly created upon the metal electrode addition). In particular, we aim to reproduce both the measured SPV and the measured QFLS in the films. To simulate partial cell stacks the work function of the top electrode is set close to midgap with a large injection barrier and low surface recombination velocities for electrons and holes. Thus, the electrodes have a negligible impact on the device. We also note that we consider an intrinsic active layer (consistent with previous experiments^14^) therefore the *V*_BI_ drops homogeneously over the active layer, which is likely not the case as real perovskite cells which exhibit significant mobile ion densities.^40−45^ As discussed above the presence of a significant mobile ion density will lead to a narrow voltage drop over the interfaces and partially field-free regions in the bulk.

**Figure 5:**
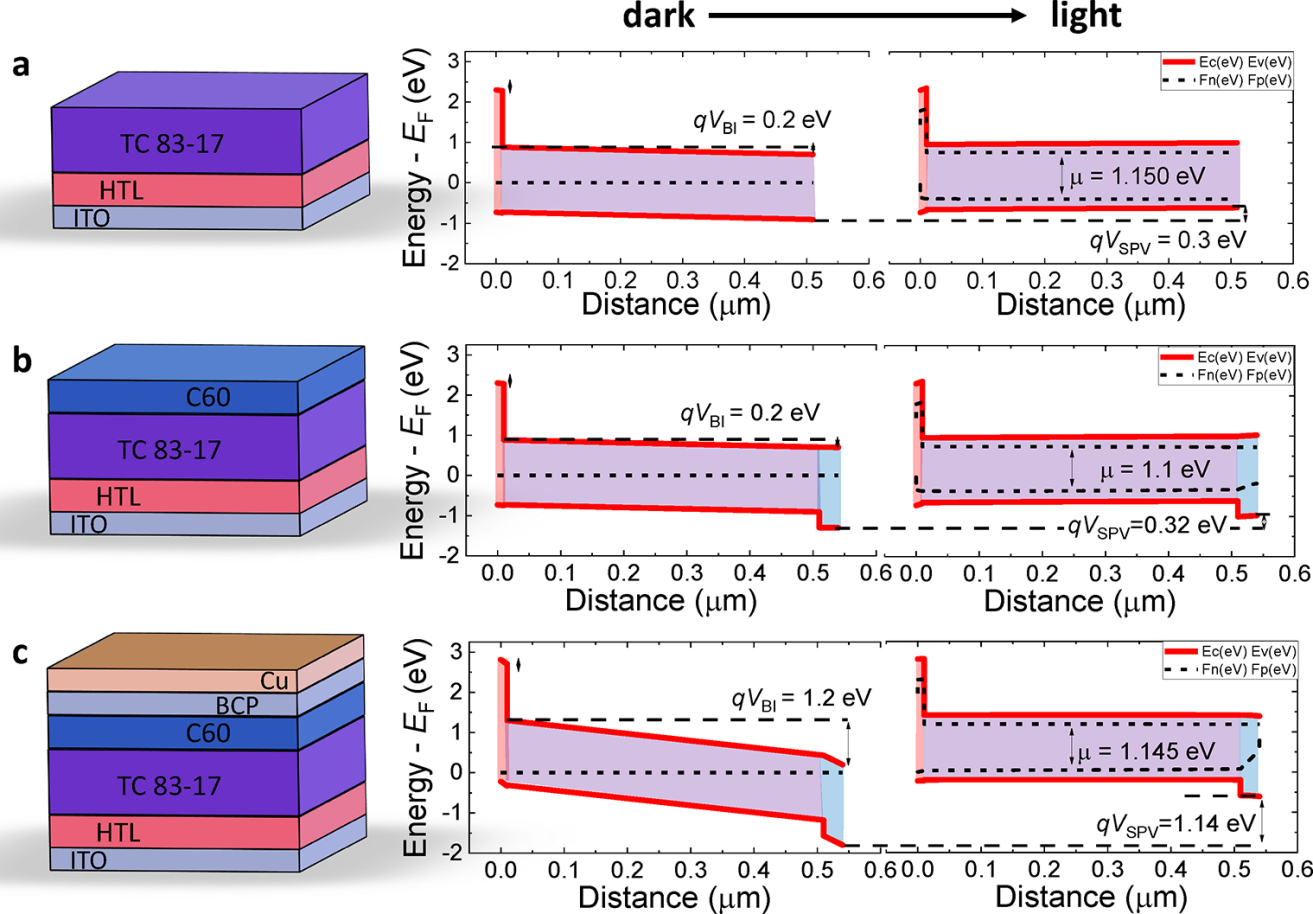
(a–c) Schematics and corresponding simulated band diagrams of different stacks under dark and light conditions. The obtained QFLS splitting and SPV values match the experimental values reasonably well. The graph highlights that in principle no large *V*_BI_ is required to obtain a large QFLS, however, in complete devices, in order to translate the QFLS to an external voltage, the *V*_BI_ needs to be large (similar to the *V*_OC_). The graph also shows that the SPV on partial cell stacks exceeds the *V*_BI_ in these films (set to 0.2 eV). The herein presented band diagrams might differ in reality due to the presence of mobile ions and interfacial phenomena such as dipoles, however, they qualitatively reproduce the relation between the SPV, QFLS, and *V*_OC_. Corresponding simulations with mobile ions are shown in Figure S12.

As shown in Figure 5, the measured SPV and the QFLS can be qualitatively reproduced for the simulations without ions, while the results for the simulations with ions (density of 5 × 10^17^ cm^–3^ for both cations and anions) are shown in Figure S13 which shows the same trends corroborating the results and following discussion. In particular, we observe that upon addition of the ETL, the SPV increases slightly due to the increased selectivity. Interestingly, because the QLFS is generated in the perovskite upon illumination (i.e., can be regarded as the internal voltage), no large *V*_BI_ is required to generate a relatively large QFLS (although a larger *V*_BI_ will also slightly increase the QFLS). Instead, the low field causes the electron and hole quasi-Fermi levels to bend toward the surface as explained in the models of Würfel and Holman and co-workers, while the QFLS stays relatively large inside the bulk.^12,46,47^ In order to translate the large internal QFLS to a large *V*_OC_ in complete devices, the electric field should ideally not change direction (relative to short-circuit) as this would push photogenerated carriers toward the opposite contact thereby usually triggering significant additional recombination of electrons at the hole contact and vice versa. To avoid reverse fields under open-circuit conditions, the *V*_OC_ should be smaller than the *V*_BI_ or at least comparable. The simulations also reveal that the SPV can easily exceed the *V*_BI_. This happens e.g., in partial cell stacks with less interface recombination (e.g., the HTL/perovskite stack). In this case, the selective charge separation leads to an inversion of the electric field (relative to 0 V). This underlines that the SPV can be larger than the *V*_BI_ and therefore, we regard the measured SPV values as upper limits of the internal field in the partial cell stacks. This is shown in the simulations in Figure S14 for the 83:17 triple cation device model with PTAA and realistic recombination parameters and relatively low SPVs (<1 V).^17^ However, for complete devices with larger SPVs (= *V*_OC_ of 1.2 V), the *V*_BI_ can exceed the SPV. Overall, the simulations rationalize the experimental findings and shed light on the subtleties of SPV and QFLS measurements in partial cell stacks.

Finally, we note that layer-by-layer SPV measurements are a powerful tool that is experimentally easily accessible similar to layer-by-layer SPV measurements. While, the exact trend between the SPV of partial cell stacks and the device performance requires further studies, to exemplify their usefulness, we simulated the SPV of HTL/perovskite with different energy alignments (Figure S15) which was motivated by a previous study.^58^ The results highlight that energetic offsets can increase the SPV (while reducing the device *V*_OC_), while energetic barriers will reduce the SPV (or even make it negative) by driving electrons to the HTL interface which compensates the flux of holes to this interface. These observations may explain the poor correlation between the SPV of HTL/perovskite stacks and the device *V*_OC_ observed and highlight how layer-by-layer SPV can be used to understand the device’s internal energetics.

## Conclusions

In summary, by studying devices with significant differences in the open-circuit voltages, we uncovered intriguing insights into the origin of the built-in voltage in perovskite solar cells by using surface photovoltage KP and UPS measurements. We first emphasize that the SPV can originate from bend bending at the bottom or the top of the cell and that SPV should be simply seen as the (hypothetical) open-circuit voltage produced by the half-stack in analogy to the cell’s *V*_OC_. For *pin*-type cells with different HTLs, we found that each successive layer of a perovskite solar cell device stack adds to the SPV when building the cell from the bottom up (ITO/HTL/perovskite/ETL/BCP/Cu). However, in well-performing cells (based on PolyTPD, PTAA, and SAM), the generated SPV in all films remains relatively low (<0.5 V) until the Cu layer is added which contributes most significantly to the SPV. This indicates that an effective electrode work function mismatch (e.g., at the inner interfaces between the electrodes and the adjacent layers) plays an important role in the *V*_BI_ in these *pin*-type cells. In the case of a 2 PACz device, the work function mismatch between 2PACz and copper amounts to approximately 1.2 eV which is close to the expected *V*_BI_ from simulations although a smaller mismatch was obtained for the other HTLs. However, it is important to note that the work function (WF) at the BCP/metal interface could be actually lower than 4 eV, as shown in the case of gold in Figure S16, which would result in a larger *V*_BI_. Using device simulations and previously established device models we find that large QFLS values (>*V*_OC_) can be sustained in the partial cells stacks without a significant internal field. The reason is that the QFLS decreases at the surface which impacts the QFLS in the bulk relatively little. However, in order to translate the large QFLS to a large *V*_OC_, a significant *V*_BI_ needs to be present in the device in the dark (roughly estimated to be >1 V for the triple cation reference cell). The study also clarifies the relationship and correlations between critical parameters, such as SPV, *V*_BI_, QFLS, and device *V*_OC_. For example, the SPV in the HTL/perovskite stack is not well correlated with the Voc of the device. This could be because energy offsets between the perovskite and the HTL reduce the *V*_OC_, while increasing the SPV. We also found that the SPV deviates from the *V*_BI_, as charge extraction alone can enhance the SPV without the need for a *V*_BI_. Perhaps, most importantly, the study highlights that the SPV of a partial cell stack is a critical and easy-to-access parameter that should be measured in addition to the QFLS of the stack. For example, these measurements may allow us to distinguish the energy alignment of different HTLs with respect to the perovskite. As such, we believe that methodology forms a solid basis for future work, especially for improving the accuracy of the simulation models. Overall, this work presents a step forward in understanding the internal junctions in perovskite cell stacks and clarifies important experimental observations with respect to SPV and QFLS measurements on partial stacks.

## Data Availability

The data that support the findings of this study are available from the corresponding author upon reasonable request.

## Supporting Information Available

Simulations of triple cation cells with only radiative recombination; simulated *JV* curves for different built-in voltages and recombination parameters; photovoltaic performance metrics for triple cation cells with different HTLs; SPV and work function measurements for different laboratories; layer-by-layer UPS-based measurements for different hole transport layers. layer-by-layer Kelvin probe measurements for different hole transport layers measured at HZB; layer-by-layer Kelvin probe measurements for different hole transport layers measured at UP; schematic band diagram showing dependence on the WF of the HTL; layer-by-layer SPV variation for well-performing devices; simulated layer-by-layer SPV and open-circuit voltage for a PEDOT:PSS transport layer; WF and *JV* comparison for devices with and without BCP layer; WF change with C60 thickness; simulated mobile ion density impact on SPV and QFLS in partial cell stacks; SPV vs. VBI in partial stacks and complete cells; simulated SPV of HTL/perovskite stacks with different energy alignment; Work function change of triple cation devices for different Au thicknesses; SCAPS simulation parameters for triple cation cells; SPV measurements for different stack layers across three labs; WF measurements for stack layers across three labs (PDF)
