# Supplementary material for: Toward Understanding the Built-in Field in Perovskite Solar Cells through Layer-by-Layer Surface Photovoltage Measurements
Source: ACS Appl Mater Interfaces. 2025 Feb 5;17(7):11176–86. doi: 10.1021/acsami.4c14194 (PMC11843610; doi:10.1021/acsami.4c14194)
Supplement: Supplementary file 1 — am4c14194_si_001.pdf [file am4c14194_si_001.pdf]

Supporting Information

Towards understanding the built-in-field in perovskite solar cells through layer-by-layer surface photovoltage measurements

Emilio Gutierrez-Partida[1], Marin Rusu[2], Fengshuo Zu[3,4], Meysam Raoufi[1], Jonas Diekmann[1], Nurlan Tokmoldin[1], Jonathan Warby[1], Dorothee Menzel[5], Felix Lang[1], Sahil Shah[1], Safa Shoaee[1], Lars Korte[5], Thomas Unold[2], Norbert Koch[3,4], Thomas Kirchartz[6,7], Dieter Neher[1], Martin Stolterfoht[1,8,*]

[1]Institute of Physics and Astronomy, University of Potsdam, Karl-Liebknecht-Str. 24-25, D-14476 Potsdam-Golm, Germany.

[2]Department Structure and Dynamics of Energy Materials, Helmholtz-Zentrum-Berlin, Hahn-Meitner-Platz 1, D-14109 Berlin, Germany

[3]Humboldt-Universitat zu Berlin, Institut für Physik & IRIS Adlershof, Brook-Taylor Straße 6, D-12489 Berlin, Germany

[4]Department Hybrid Material Systems, Helmholtz-Zentrum Berlin, Albert-Einstein-Straße 15, 12489 Berlin.

[5]Department Perovskite Tandem Solar Cells, Helmholtz-Zentrum-Berlin, Kekuléstr. 5, D-12489 Berlin, Germany

[6]IEK5-Photovoltaik, IEK5-Photovoltaik, Forschungszentrum Jülich GmbH, Wilhelm-Johnen-Straße, 52428 Jülich, Germany.

[7]Faculty of Engineering and CENIDE, University of Duisburg-Essen, Carl-Benz-Str. 199, 47057 Duisburg, Germany

[8]Electronic Engineering Department, The Chinese University of Hong Kong, Sha Tin N.T., Hong Kong SAR, China

*email: mstolterfoht@ee.cuhk.edu.hk

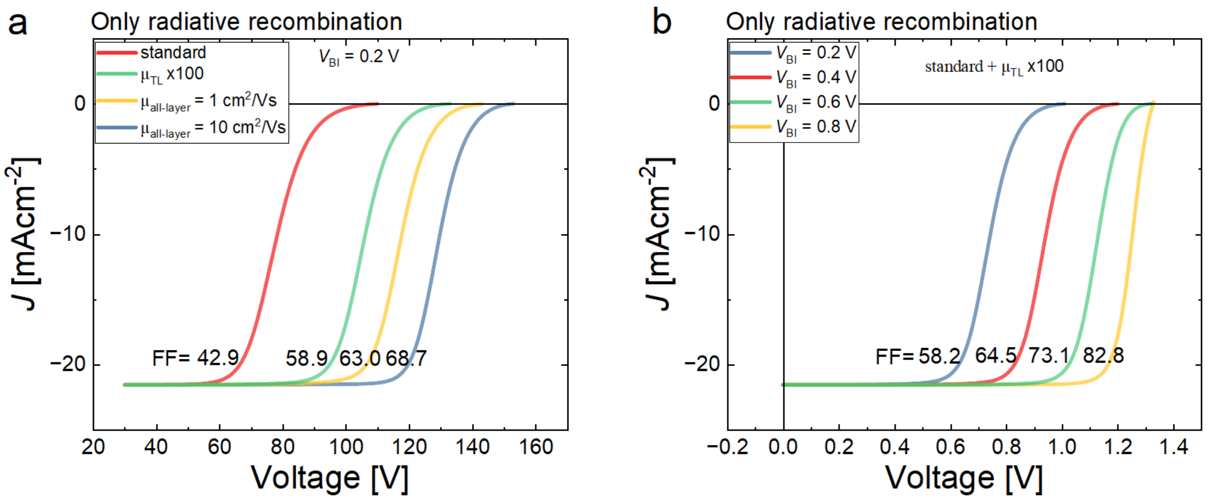

Figure S1. a Simulated *JV*s curves starting from the standard device model for triple cation cells (**Table S1**) but without non-radiative recombination (radiative-only) and a low built-in voltage of 0.2 V. Even when the mobilities are increased in all layers, the experimental fill factor (FF) of 80% cannot be reproduced. **b** Simulated *JV*s curves for triple cation cells with radiative recombination only and 100x higher mobilities in the transport layers, demonstrating that a V_{BI} of roughly 0.8 eV is still insufficient to reproduce the measured FF. The reason for the FF loss is increased radiative recombination under the reverse internal field when the voltage exceeds the V_{BI}.

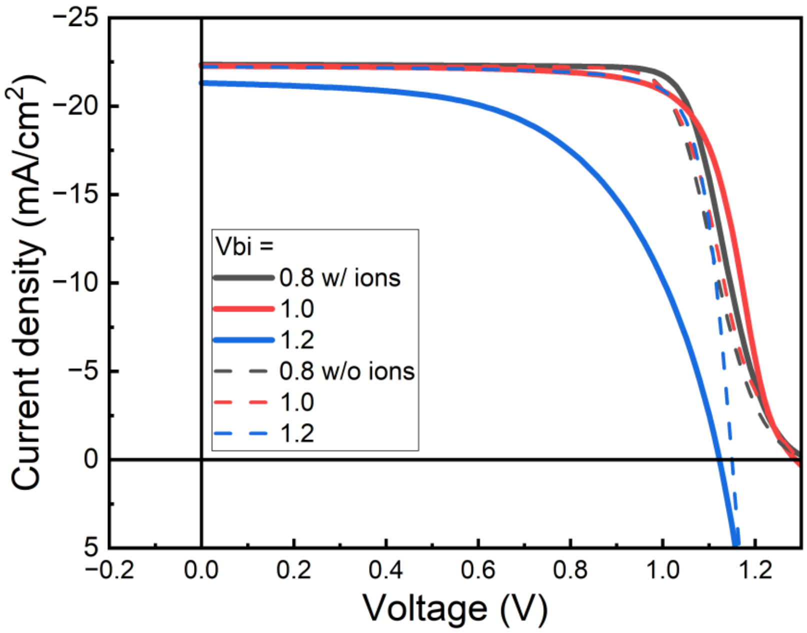

Figure S2. Simulations of a standard triple cation cell are shown in blue, and two cells with optimized recombination parameters (S = 10 cm s^{-1} at both interfaces and a bulk-lifetime, τ_{bulk}, of 10 µs) but a lower built-in field shown in black and red, all cases with and without mobile ions (cation densities of 5x10^{17} cm^{-3}, immobilized anions with same density). The presence of ions does not make the cells much more efficient for low V_{BI}s, and even if so the effect is small further proving the statement that the device needs to have a V_{BI} > 1V even with mobile ions.

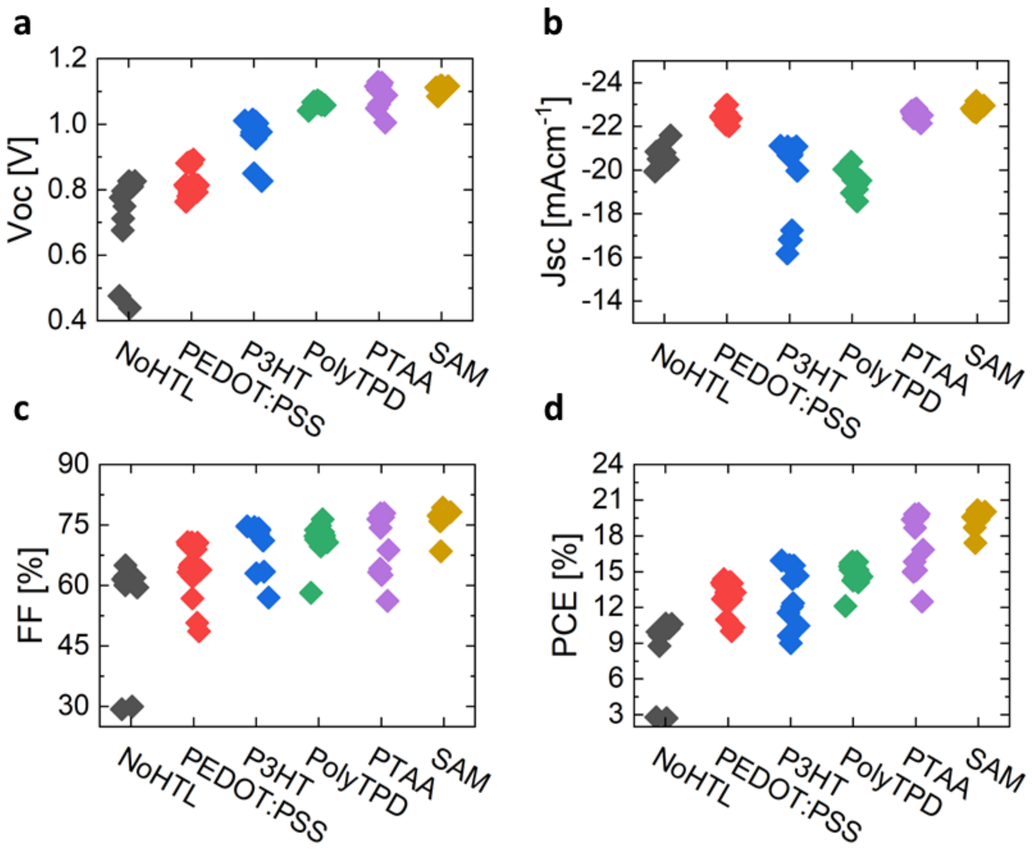

Figure S3. **a** Open-circuit voltage (V_{OC}), **b** short-circuit current density (J_{SC}), **c** fill factor (FF), and **d** the power conversion efficiency (PCE) from current-voltage characteristics measured in reverse scan at 67 mV/s on $Cs_{0.05}(FA_{0.83}MA_{0.05})_{0.95}Pb(I_{0.83}Br_{0.17})_3$ triple cation perovskite solar cells.

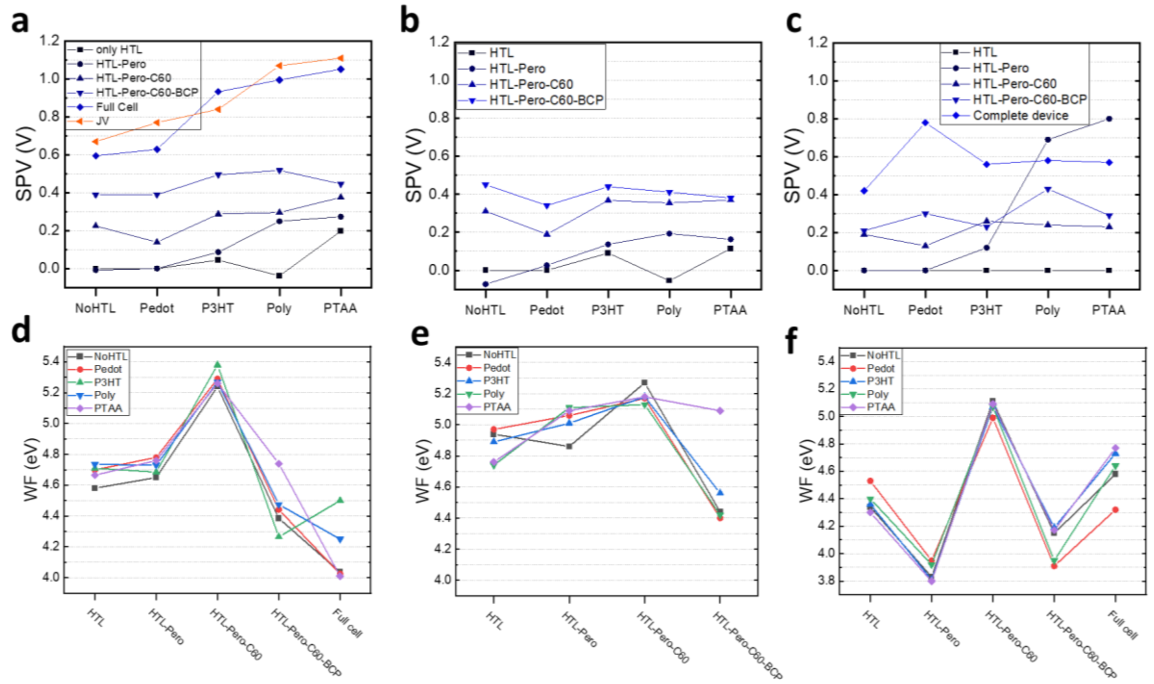

Figure S4. Comparison layer-by-layer surface photovoltage (SPV) **a,b,c** and workfunction (WF) measurements **d,e,f** in different labs; **a,d** shows measurements from Kelvin probe (KP) taken at the University of Potsdam; **b,e** shows measurements from KP taken at the Helmholtz-Zentrum Berlin, **c,f** shows measurements from ultraviolet photoelectron spectroscopy (UPS) taken at the Humboldt University Berlin. The UV light used for the UPS measurement might damage the sample as previously reported.[2] Moreover, the ultrahigh vacuum might, potentially in combination with the UV illumination, be a problem. Although the origin of the effect is not clear yet, its like that UPS shows an artificially high SPV value in case of the HTL-only sample with PTAA. This is consistent with the low SPV obtained in the devices, which might indicate sample damage. However, there is also another factor that should be considered. Given that UPS is measured with (UV) light, even at low intensities such as 1/1000th of a sun, this would already produce a significant SPV. Adding a 1 sun equivalent background illumination might therefore not result in the complete SPV, which can only be obtained with KP.

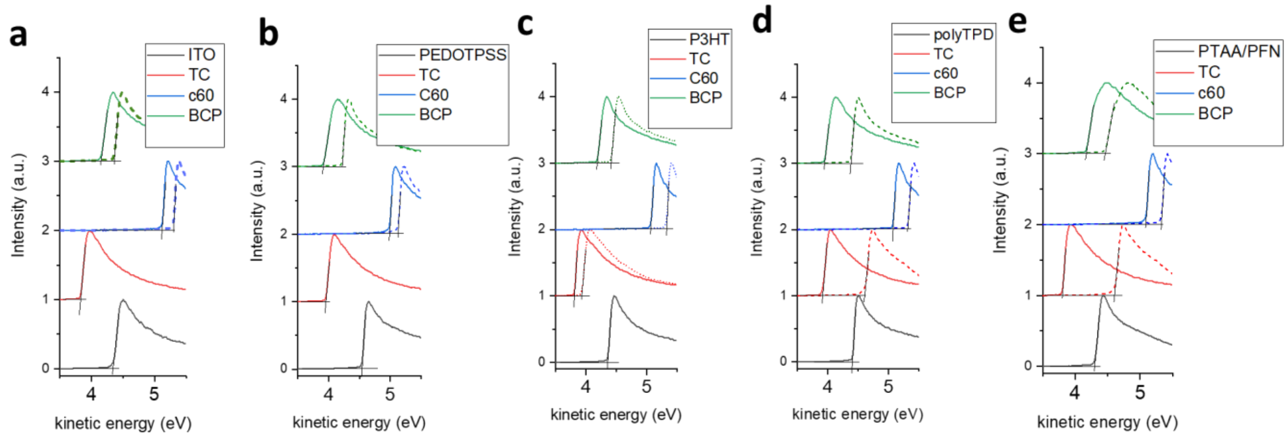

Figure S5. Layer-by-layer ultraviolet photon-electron spectroscopy (UPS) measurements taken on perovskite cells with different hole transport layers, **a** No HTL, **b** PEDOT:PSS, **c** P3HT, **d** PolyTPD, **e** PTAA/PFN as measured at Humboldt University Berlin.

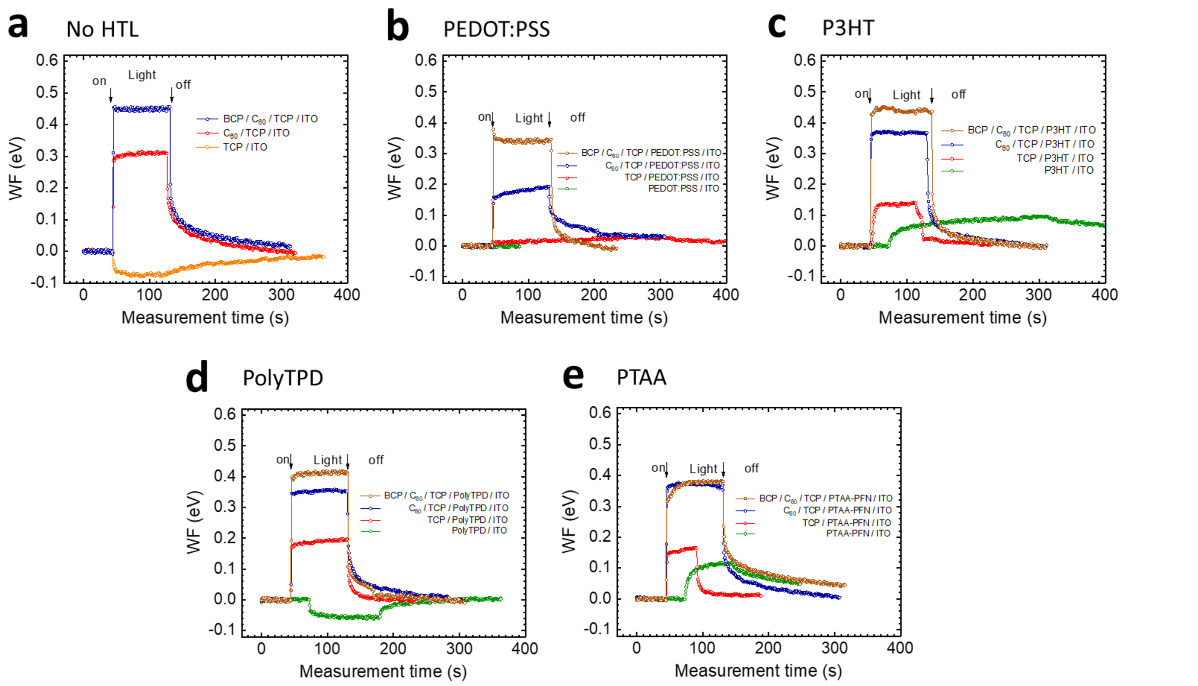

Figure S6. Layer-by-layer measurements with Kelvin Probe taken on perovskite cells with different hole transport layers **a** No HTL, **b** PEDOT:PSS, **c** P3HT, **d** PolyTPD, **e** PTAA/PFN as measured at Helmholtz-Zentrum Berlin.

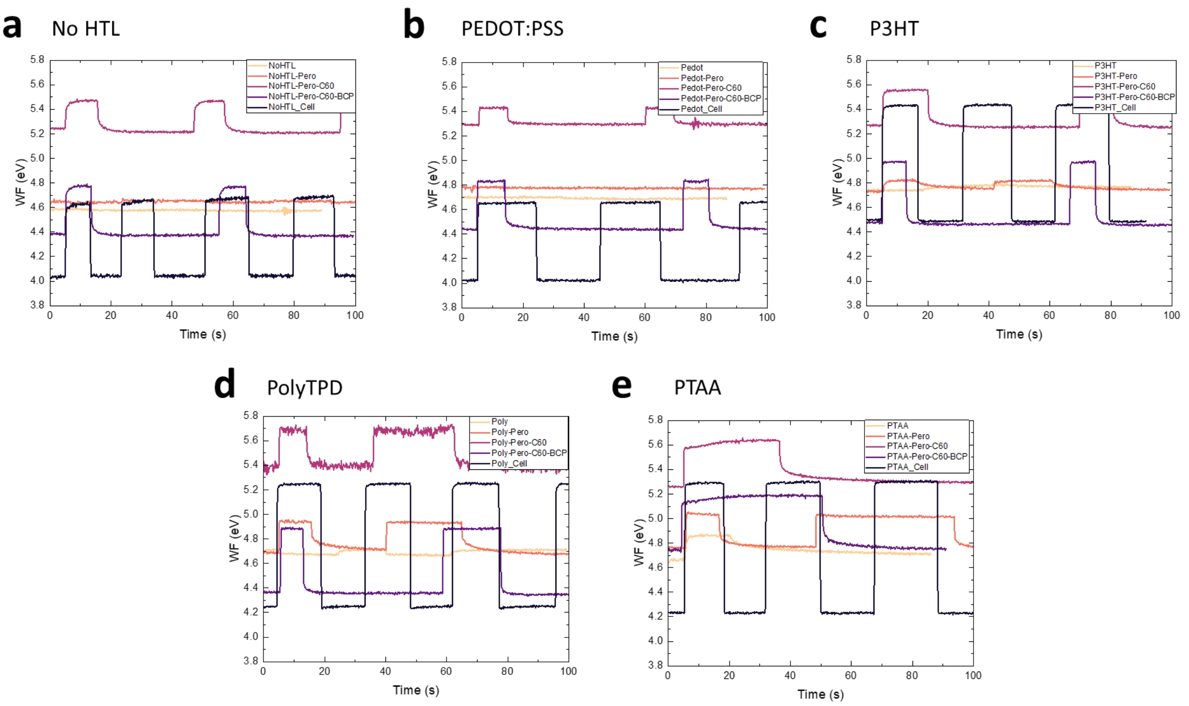

Figure S7. Layer-by-layer measurements with Kelvin Probe taken on perovskite cells with different hole transport layers **a** No HTL, **b** PEDOT:PSS, **c** P3HT, **d** PolyTPD, **e** PTAA/PFN asmeasured at Uni Potsdam.

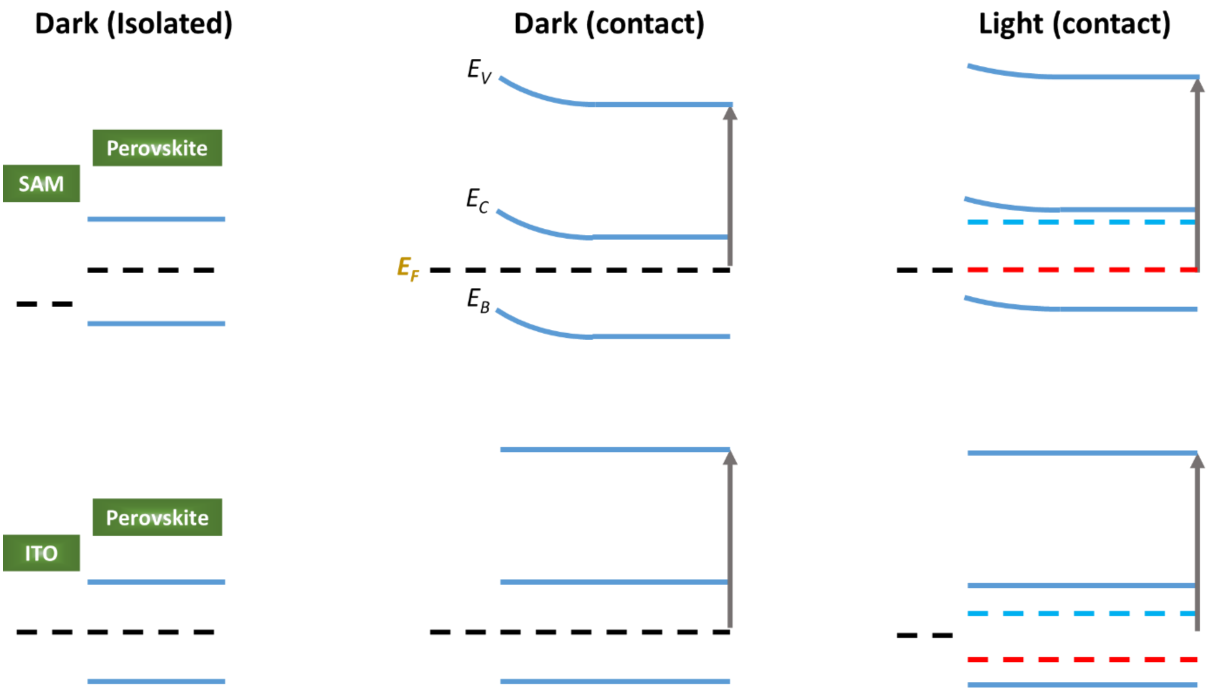

Figure S8. Schematic band diagram in the dark and in the light demonstrating how a transport layer with a large work function can cause a large SPV, while a transport layer/contact with a workfunction that is in the middle of the perovskite bandgap will likely not lead to an SPV. This could be the case with the ITO/perovskite stack.

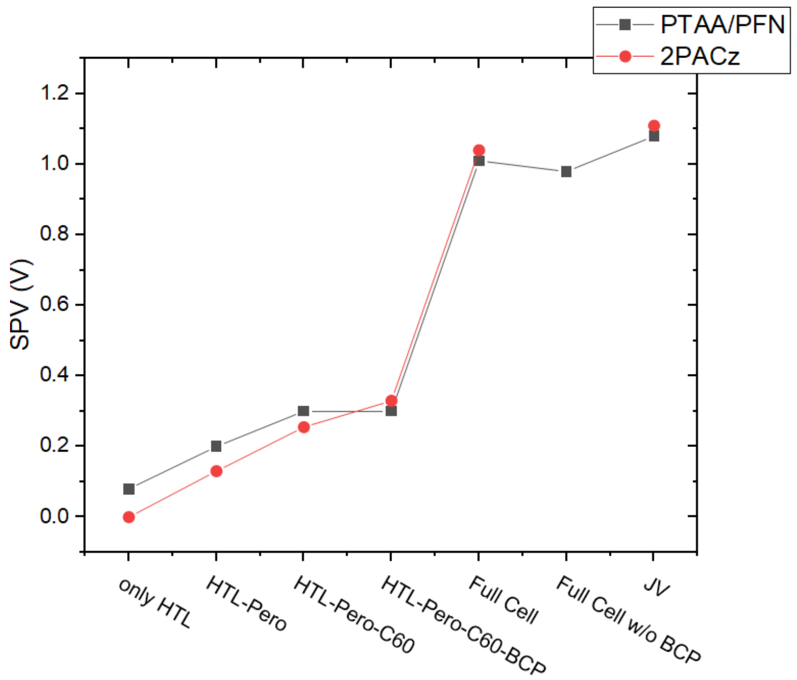

Figure S9. Change of SPV for well-performing devices.

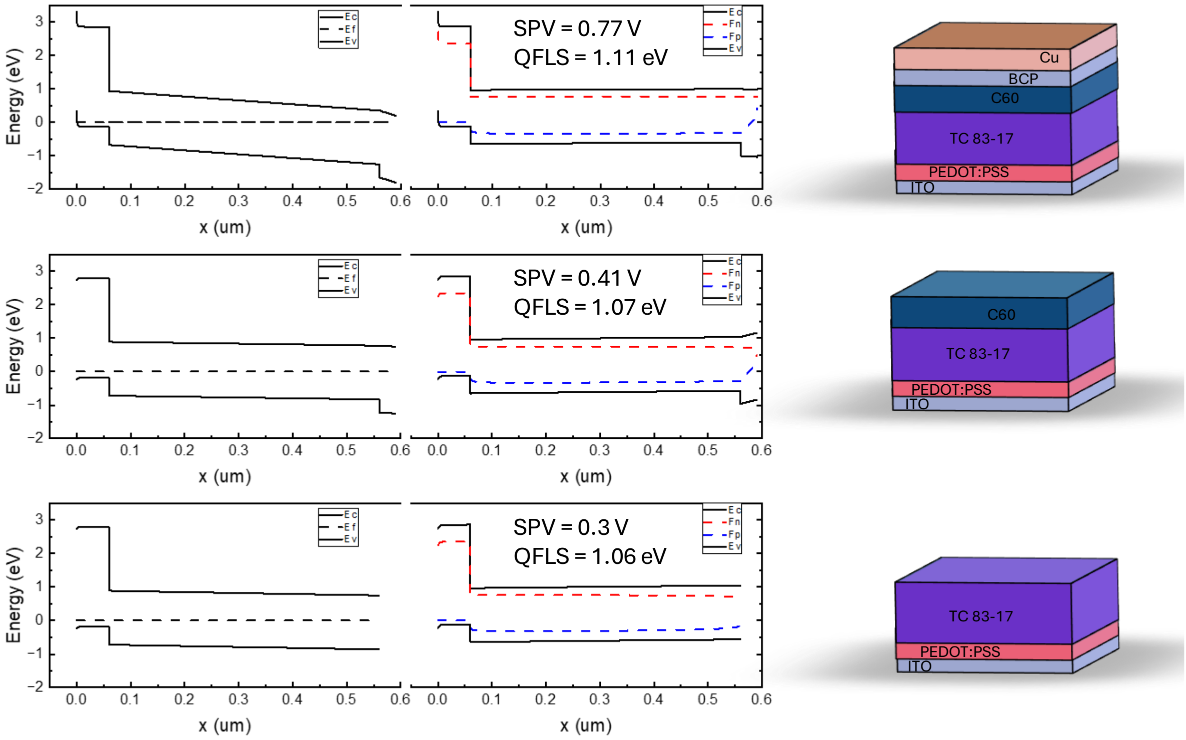

Figure S10. Impact of energy offset between PEDOT:PSS and TC 83:17 on the V_{OC} and the SPV demonstrated by SCAPS simulations. A V_{BI} of 0.2 V was assumed for the partial cell stacks.

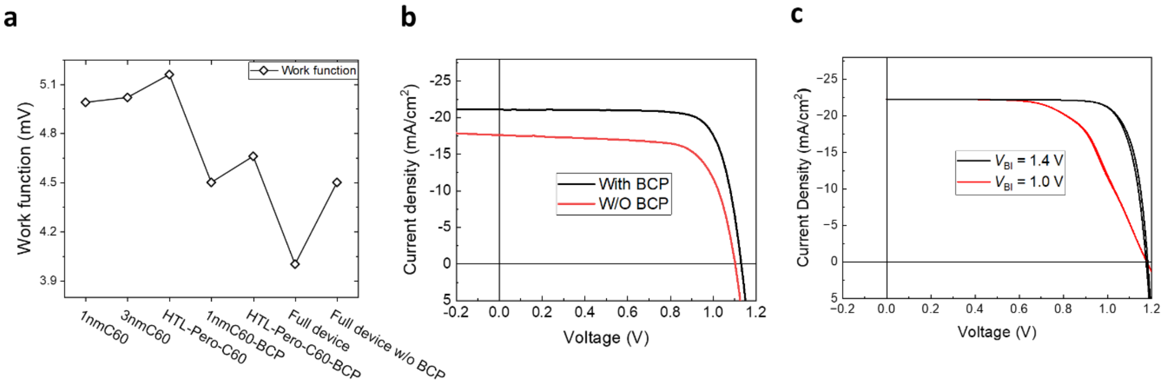

Figure S11. a Layer-by-layer work function change of devices with and w/o BCP. **b** Corresponding *JV*-curves of the devices with and w/o BCP. **c** Simulated *JV* curves for a complete device without BCP using a V_{BI} of 1.0 V (red) and a standard device with a larger V_{BI} of 1.4 V (black). We note, the experimental *JV*s of BCP could not be exactly reproduced meaning there is likely another factor that influences the performance when BPC is left out affecting particularly the J_{SC}.

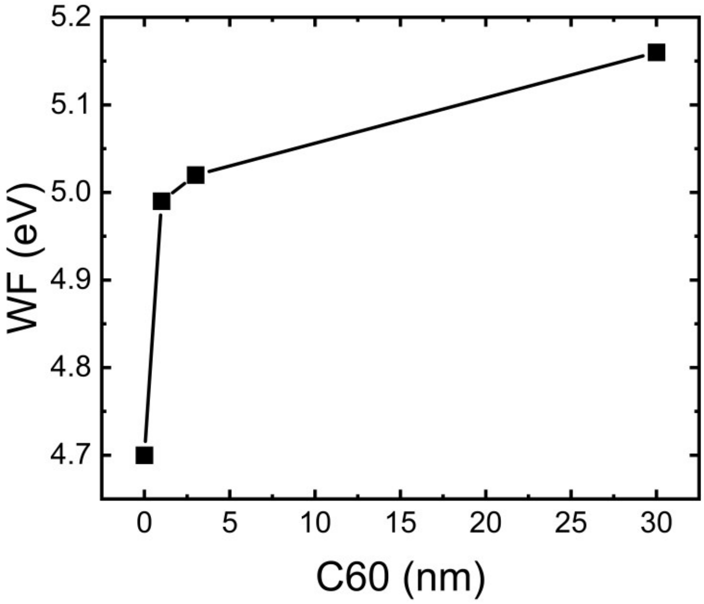

Figure S12. Work function change as a function of C_{60}-thickness.

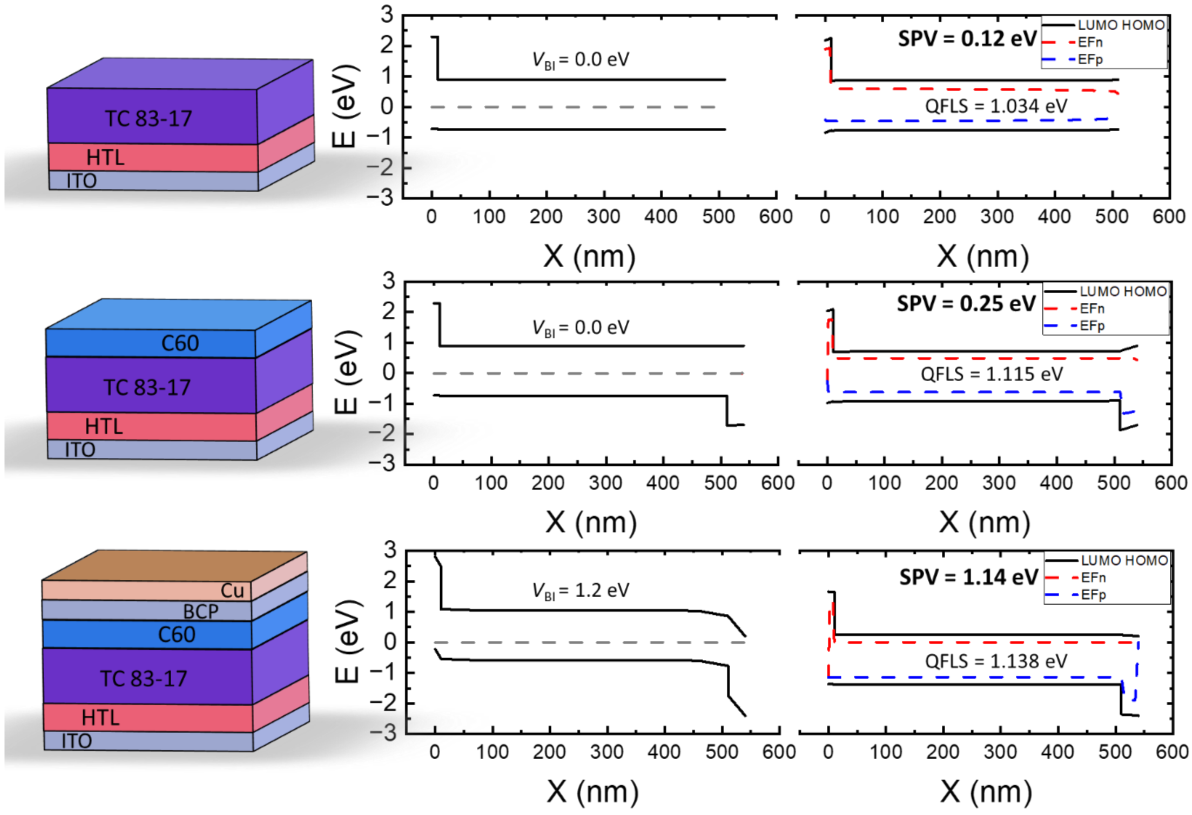

Figure S13. Impact of the mobile ions (mobile anions and cations with a density of 10^{17} cm^{-3} and constant mobility of 10^{-7} cm^2/Vs, respectively) on the SPV and QFLS of partial cell stacks considering a negligible V_{BI} (0 V) in analogy to the simulations in **Figure 5**. As expected, mobile ions shorten the depletion width, however, they do not influencing the observed trends without ions.

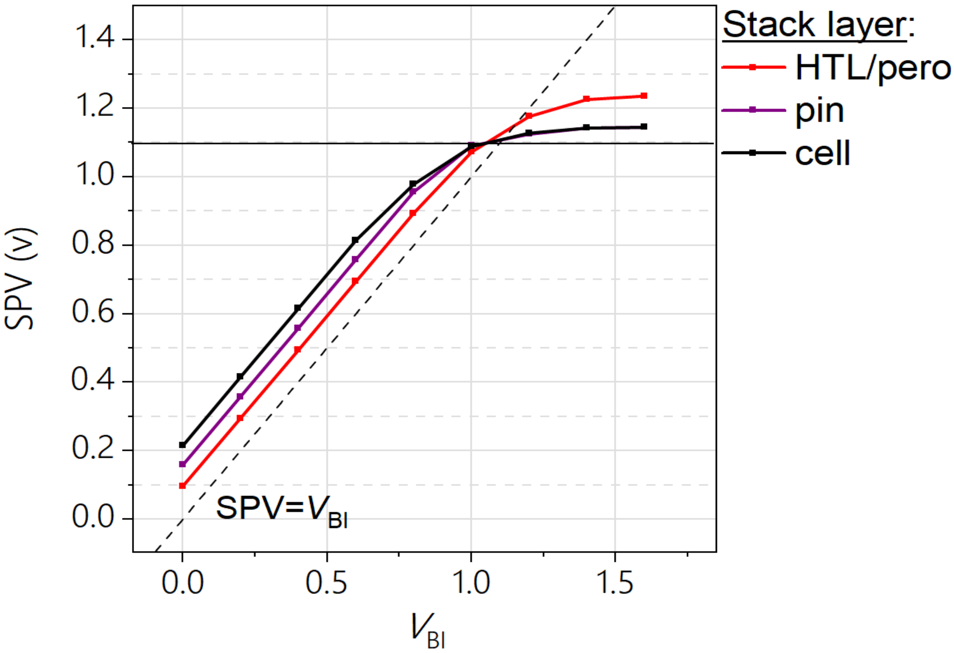

Figure S14. Device model with realistic recombination parameters and large V_{BI}. If there is a large internal voltage present in the partial cell stack, a large SPV would be obtained. Therefore, we consider the SPV to be the upper limit of the V_{BI} in partial cell stacks and the lower limit of the V_{BI} in complete cells.

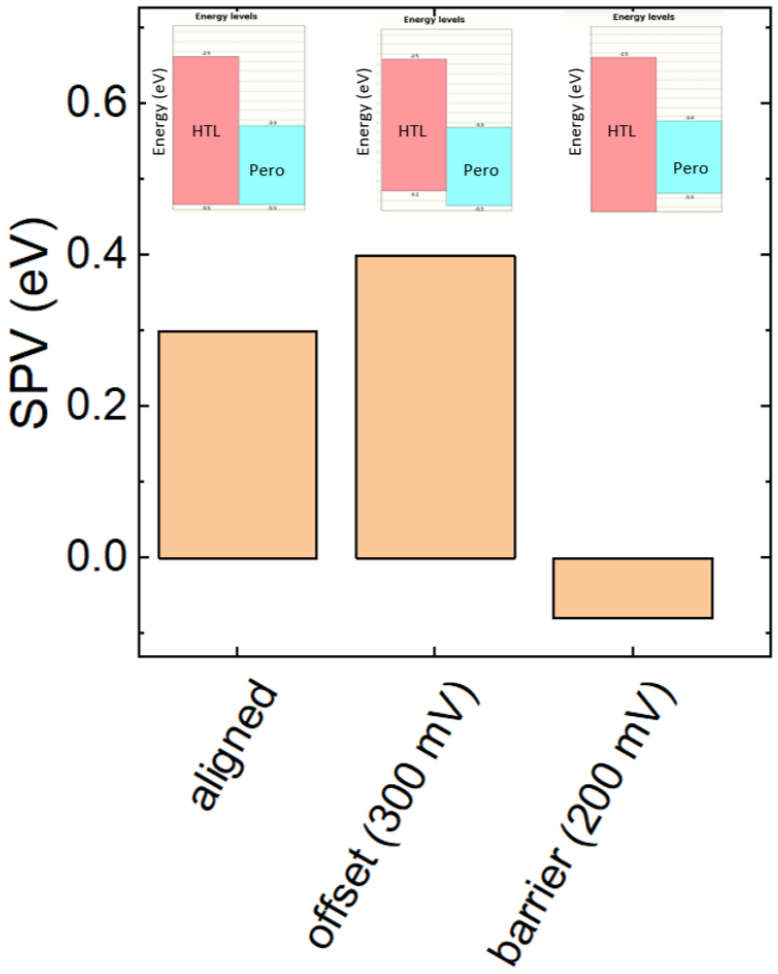

Figure S15. Simulated SPV of HTL/perovskite stacks with a PTAA layer with different energy level alignments and a minority carrier recombination velocity of 100 cm/s. An energy offset increases the SPV while a barrier reduces it.

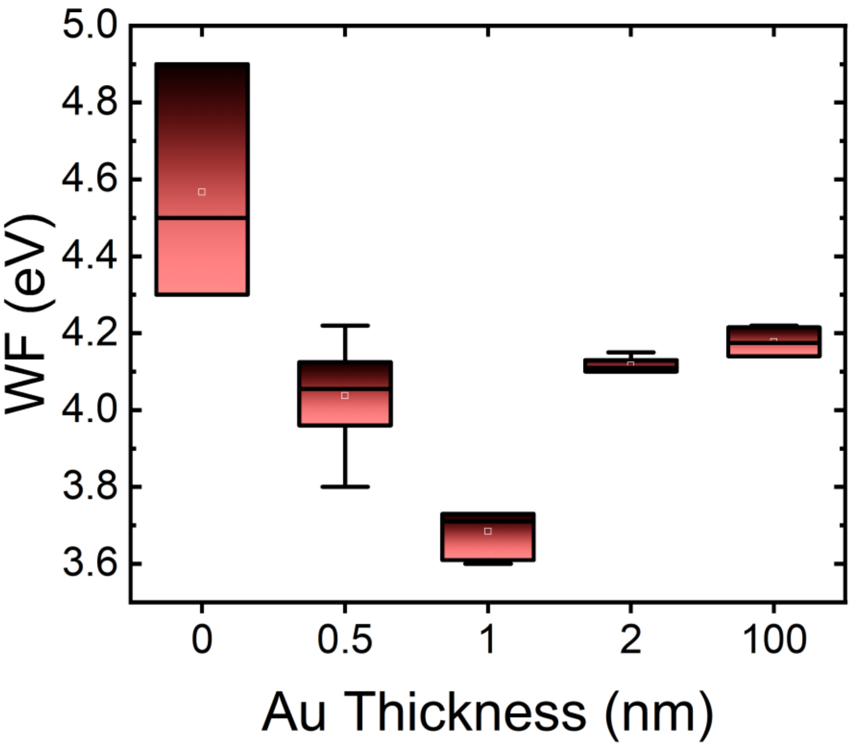

Figure S16. Work function change of triple cation devices as a function of the gold electrode thickness.

Table S1 The simulation parameters used for SCAPS for the 83:17 triple cation standard cell.

Parameter	Symbol	Value	Unit	Ref.
Majority carrier band offset between perovskite and C_{60}	$\Delta E_{\mathrm{maj,c}}$	0	eV	[3]
Majority carrier band offset between perovskite and PTAA	$\Delta E_{\mathrm{maj,v}}$	0	eV	[3]
Lifetime in perovskite	τ_{pero}	500	ns	[4]
Ionized acceptors in PTAA	$N_{\mathrm{A},p}^{-}$	0	cm^{-3}	
Ionized donors in C_{60}	$N_{\mathrm{D},n}^{+}$	0	cm^{-3}	
Minority carrier recombination velocity from perovskite to PTAA	$S_{\mathrm{min},n}$	500	cm/s	[4]
Minority carrier recombination velocity from perovskite to C_{60}	$S_{\mathrm{min},p}$	2000	cm/s	[4]
Thickness of PTAA	d_{PTAA}	10	nm	
Thickness of perovskite	d_{pero}	400	nm	
Thickness of C_{60}	d_{C60}	30	nm	
Offset between metal and PTAA	$\Delta E_{\mathrm{F,metal-}}$	0.05	eV	
Offset between metal and C_{60}	$\Delta E_{\mathrm{F,metal-}}$	0.05	eV	
Device built-in voltage	V_{BI}	1.5	V	
Bandgap PTAA	$E_{\mathrm{G,PTAA}}$	3.0	eV	
Electron affinity PTAA	$E_{\mathrm{A,PTAA}}$	2.5	eV	

Bandgap perovskite	$E_{\mathrm{G,pero}}$	1.63	eV	
Electron affinity perovskite	$E_{\mathrm{A,pero}}$	3.9	eV	[3]
Electron affinity C_{60}	$E_{\mathrm{A,C60}}$	3.9	eV	
Bandgap C_{60}	$E_{\mathrm{G,C60}}$	2.0	eV	
Electron mobility in C_{60}	$\mu_{n,\mathrm{C60}}$	$1\text{x}10^{-2}$	cm^2/Vs	[5]
Hole mobility in PTAA	$\mu_{p,\mathrm{PTAA}}$	$1\text{x}10^{-4}$	cm^2/Vs	[6]
Electron mobility in perovskite	$\mu_{n,\mathrm{pero}}$	1	cm^2/Vs	Oxford ref.
Hole mobility in perovskite	$\mu_{p,\mathrm{pero}}$	1	cm^2/Vs	
Relative dielectric constant PTAA	ϵ_{PTAA}	3.5		[7]
Relative dielectric constant perovskite	ϵ_{pero}	22		[8]
relative dielectric constant C_{60}	ϵ_{C60}	5.0		[9]
Effective electron density of states in HTL	$N_{\mathrm{C/V,PTAA}}$	$1\text{x}10^{20}$	cm^{-3}	[10]
Effective electron density of states in C_{60}	$N_{\mathrm{C/V,C60}}$	$1\text{x}10^{20}$	cm^{-3}	[10]
Effective electron density of states in perovskite	$N_{\mathrm{C/V,pero}}$	$2.2\text{x}10^{18}$	cm^{-3}	[11]
Effective ion density	N_{ion}	variable	cm^{-3}	
Effective ion diffusion constant	D_{ion}	$5\text{x}10^{-10}$	cm^2/s	

Table S2. SPV measured by Kelvin probe of various stack layers of different cells measured at University of Potsdam (UP), Helmholtz Zentrum Berlin (HZB) and Humboldt University (HU).

UP		NoHTL	Pedot	P3HT	Poly	PTAA	SAM 2Pacz
SPV [V] from KP	HTL	0	0	0.045	-0.038	0.197	0
	HTL-Pero	-0.008	0	0.087	0.249	0.273	0.13
	HTL-Pero-C60	0.225	0.139	0.288	0.296	0.376	0.26
	HTL-Pero-C60-BCP	0.389	0.391	0.495	0.518	0.446	0.33
	Complete device	0.595	0.629	0.933	0.995	1.052	1.04
Voc [V]	JV	0.67	0.77	0.84	1.07	1.11	1.11
C vs V peak [V]	C-V	0.74	0.71	1.01	1.08	1.06	-

HZB		NoHTL	Pedot	P3HT	Poly	PTAA
SPV [V] from KP	HTL	0	0	0.091	-0.055	0.114
	HTL-Pero	-0.073	0.026	0.137	0.193	0.163
	HTL-Pero-C60	0.311	0.190	0.368	0.355	0.370
	HTL-Pero-C60-BCP	0.451	0.341	0.440	0.412	0.380
	Complete device	0.686	0.766	0.956	1.024	1.045

HU		NoHTL	Pedot	P3HT	Poly	PTAA
SPV [V] from UPS	HTL	0	0	0	0	0
	HTL-Pero	0	0	0.12	0.69	0.8
	HTL-Pero-C60	0.19	0.13	0.26	0.24	0.23
	HTL-Pero-C60-BCP	0.21	0.3	0.23	0.43	0.29
	Complete device	0.42	0.78	0.56	0.58	0.57

Table S3. Work function measured by Kelvin probe of various stack layers of different cells measured at University of Potsdam (UP), Helmholtz Zentrum Berlin (HZB) and Humboldt University (HU).

UP	WF in the dark [eV]				
	HTL	HTL-Pero	HTL-Pero-C60	HTL-Pero-C60-BCP	Complete device
NoHTL	4.58	4.65	5.24	4.385	4.036
Pedot	4.7	4.78	5.29	4.441	4.025
P3HT	4.71	4.685	5.38	4.265	4.5
Poly	4.735	4.73	5.27	4.475	4.25
PTAA	4.665	4.76	5.26	4.74	4.233
SAM 2Pacz	5.2	5.01	5.18	4.55	4.01

HZB	WF in the dark [eV]			
	HTL	HTL-Pero	HTL-Pero-C60	HTL-Pero-C60-BCP
NoHTL	4.94	4.86	5.27	4.44
Pedot	4.97	5.06	5.17	4.40
P3HT	4.89	5.01	5.18	4.56
Poly	4.74	5.11	5.13	4.42
PTAA	4.76	5.09	5.18	5.09

HU	WF in the dark [eV]				
	HTL	HTL-Pero	HTL-Pero-C60	HTL-Pero-C60-BCP	Complete device
NoHTL	4.34	3.83	5.11	4.15	4.58
Pedot	4.53	3.95	4.99	3.91	4.32
P3HT	4.36	3.81	5.07	4.19	4.73
Poly	4.4	3.92	5.07	3.05	4.64
PTAA	4.3	3.8	5.09	4.17	4.77

References

(1) Diekmann, J.; Caprioglio, P.; Futscher, M. H.; Le Corre, V. M.; Reichert, S.; Jaiser, F.; Arvind, M.; Toro, L. P.; Gutierrez-Partida, E.; Peña-Camargo, F.; Deibel, C.; Ehrler, B.; Unold, T.; Kirchartz, T.; Neher, D.; Stolterfoht, M. Pathways toward 30% Efficient Single-Junction Perovskite Solar Cells and the Role of Mobile Ions. *Solar RRL* **2021**, *5* (8). https://doi.org/10.1002/solr.202100219.

(2) Hu, J.; Chen, P.; Luo, D.; Wang, D.; Chen, N.; Yang, S.; Fu, Z.; Yu, M.; Li, L.; Zhu, R.; Lu, Z. H. Tracking the Evolution of Materials and Interfaces in Perovskite Solar Cells under an Electric Field. *Commun Mater* **2022**, *3* (1). https://doi.org/10.1038/s43246-022-00262-2.

(3) Stolterfoht, M.; Caprioglio, P.; Wolff, C. M.; Márquez, J. A.; Nordmann, J.; Zhang, S.; Rothhardt, D.; Hörmann, U.; Amir, Y.; Redinger, A.; Kegelmann, L.; Zu, F.; Albrecht, S.; Koch, N.; Kirchartz, T.; Saliba, M.; Unold, T.; Neher, D. The Impact of Energy Alignment and Interfacial Recombination on the Internal and External Open-Circuit Voltage of Perovskite Solar Cells. *Energy Environ Sci* **2019**, *12* (9), 2778–2788. https://doi.org/10.1039/C9EE02020A.

(4) Stolterfoht, M.; Wolff, C. M.; Márquez, J. A.; Zhang, S.; Hages, C. J.; Rothhardt, D.; Albrecht, S.; Burn, P. L.; Meredith, P.; Unold, T.; Neher, D. Visualization and Suppression of Interfacial Recombination for High-Efficiency Large-Area Pin Perovskite Solar Cells. *Nat Energy* **2018**, *3* (10), 847–854. https://doi.org/10.1038/s41560-018-0219-8.

(5) Mendil, N.; Daoudi, M.; Berkai, Z.; Belghachi, A. Disorder Effect on Carrier Mobility in Fullerene Organic Semiconductor. *J Phys Conf Ser* **2015**, *647* (1), 012057. https://doi.org/10.1088/1742-6596/647/1/012057.

(6) Luo, J.; Xia, J.; Yang, H.; Chen, L.; Wan, Z.; Han, F.; Malik, H. A.; Zhu, X.; Jia, C. Toward High-Efficiency, Hysteresis-Less, Stable Perovskite Solar Cells: Unusual Doping of a Hole-Transporting Material Using a Fluorine-Containing Hydrophobic Lewis Acid. *Energy Environ Sci* **2018**, *11* (8), 2035–2045. https://doi.org/10.1039/c8ee00036k.

(7) Torabi, S.; Jahani, F.; Van Severen, I.; Kanimozhi, C.; Patil, S.; Havenith, R. W. A.; Chiechi, R. C.; Lutsen, L.; Vanderzande, D. J. M.; Cleij, T. J.; Hummelen, J. C.; Koster, L. J. A. Strategy for Enhancing the Dielectric Constant of Organic Semiconductors without Sacrificing Charge Carrier Mobility and Solubility. *Adv Funct Mater* **2015**, *25* (1), 150–157. https://doi.org/10.1002/adfm.201402244.

(8) Brivio, F.; Butler, K. T.; Walsh, A.; Van Schilfgaarde, M. Relativistic Quasiparticle Self-Consistent Electronic Structure of Hybrid Halide Perovskite Photovoltaic Absorbers. *Phys Rev B Condens Matter Mater Phys* **2014**, *89* (15), 1–6. https://doi.org/10.1103/PhysRevB.89.155204.

(9) Chern, G.; Mathias, H.; Testardi, L. R.; Seger, L.; Schlenoff, J. Low-Frequency Dielectric Permittivity of C60. *Journal of Superconductivity* **1995**, *8* (2), 207–210. https://doi.org/10.1007/BF00732372.

(10) Kirchartz, T.; Gong, W.; Hawks, S. A.; Agostinelli, T.; MacKenzie, R. C. I.; Yang, Y.; Nelson, J. Sensitivity of the Mott–Schottky Analysis in Organic Solar Cells. *The Journal of Physical Chemistry C* **2012**, *116* (14), 7672–7680.

(11) Staub, F.; Hempel, H.; Hebig, J.-C.; Mock, J.; Paetzold, U. W.; Rau, U.; Unold, T.; Kirchartz, T. Beyond Bulk Lifetimes: Insights into Lead Halide Perovskite Films from Time-Resolved
